# Different SWI/SNF complexes coordinately promote R-loop- and RAD52-dependent transcription-coupled homologous recombination

**DOI:** 10.1093/nar/gkad609

**Published:** 2023-07-20

**Authors:** Carlota Davó-Martínez, Angela Helfricht, Cristina Ribeiro-Silva, Anja Raams, Maria Tresini, Sidrit Uruci, Wiggert A van Cappellen, Nitika Taneja, Jeroen A A Demmers, Alex Pines, Arjan F Theil, Wim Vermeulen, Hannes Lans

**Affiliations:** Department of Molecular Genetics, Erasmus MC Cancer Institute, Erasmus University Medical Center, Rotterdam 3015 GD, The Netherlands; Department of Molecular Genetics, Erasmus MC Cancer Institute, Erasmus University Medical Center, Rotterdam 3015 GD, The Netherlands; Department of Molecular Genetics, Erasmus MC Cancer Institute, Erasmus University Medical Center, Rotterdam 3015 GD, The Netherlands; Department of Molecular Genetics, Erasmus MC Cancer Institute, Erasmus University Medical Center, Rotterdam 3015 GD, The Netherlands; Department of Molecular Genetics, Erasmus MC Cancer Institute, Erasmus University Medical Center, Rotterdam 3015 GD, The Netherlands; Department of Molecular Genetics, Erasmus MC Cancer Institute, Erasmus University Medical Center, Rotterdam 3015 GD, The Netherlands; Erasmus Optical Imaging Center, Erasmus University Medical Center, Rotterdam 3015 GD, The Netherlands; Department of Molecular Genetics, Erasmus MC Cancer Institute, Erasmus University Medical Center, Rotterdam 3015 GD, The Netherlands; Proteomics Center, Erasmus University Medical Center, Rotterdam 3015 GD, The Netherlands; Department of Molecular Genetics, Erasmus MC Cancer Institute, Erasmus University Medical Center, Rotterdam 3015 GD, The Netherlands; Department of Molecular Genetics, Erasmus MC Cancer Institute, Erasmus University Medical Center, Rotterdam 3015 GD, The Netherlands; Department of Molecular Genetics, Erasmus MC Cancer Institute, Erasmus University Medical Center, Rotterdam 3015 GD, The Netherlands; Department of Molecular Genetics, Erasmus MC Cancer Institute, Erasmus University Medical Center, Rotterdam 3015 GD, The Netherlands

## Abstract

The SWI/SNF family of ATP-dependent chromatin remodeling complexes is implicated in multiple DNA damage response mechanisms and frequently mutated in cancer. The BAF, PBAF and ncBAF complexes are three major types of SWI/SNF complexes that are functionally distinguished by their exclusive subunits. Accumulating evidence suggests that double-strand breaks (DSBs) in transcriptionally active DNA are preferentially repaired by a dedicated homologous recombination pathway. We show that different BAF, PBAF and ncBAF subunits promote homologous recombination and are rapidly recruited to DSBs in a transcription-dependent manner. The PBAF and ncBAF complexes promote RNA polymerase II eviction near DNA damage to rapidly initiate transcriptional silencing, while the BAF complex helps to maintain this transcriptional silencing. Furthermore, ARID1A-containing BAF complexes promote RNaseH1 and RAD52 recruitment to facilitate R-loop resolution and DNA repair. Our results highlight how multiple SWI/SNF complexes perform different functions to enable DNA repair in the context of actively transcribed genes.

## Graphical Abstract

**Graphical Abstract:**
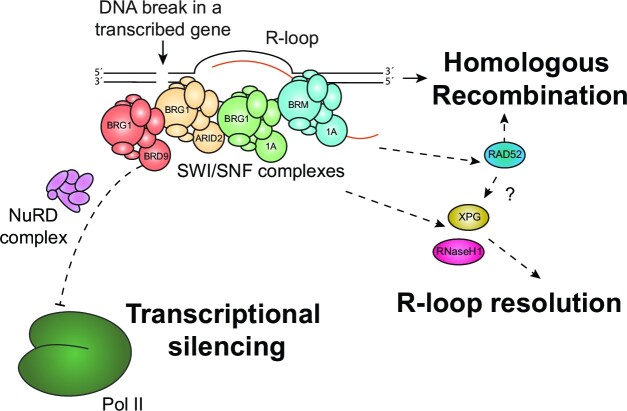

## INTRODUCTION

DNA is continuously damaged by numerous environmental and cell-intrinsic genotoxic agents. Double-strand breaks (DSBs) are one of the most deleterious forms of DNA damage and can lead to genomic rearrangements or mutations if not adequately repaired. Cells have developed multiple DNA repair mechanisms to deal with various types of DNA damage, including DSBs for which mainly non-homologous end joining (NHEJ) and homologous recombination (HR) are used (1,2). Classical HR is the prevalent DSB repair mechanism in the S/G2 phase of the cell cycle, in which it uses the sister chromatid as homologous template for error-free repair. HR is initiated by DNA end-resection mediated by the MRN complex, together with CtIP, to generate single-stranded DNA (ssDNA), which is followed by more extensive DNA end-resection by the EXO1 and DNA2 nucleases. The RPA complex binds to the ssDNA and, with the help of multiple proteins including BRCA2, is exchanged for the RAD51 recombinase that facilitates homologous strand invasion and repair. Some DNA repair pathways are dependent on active transcription, such as, for instance, transcription-coupled nucleotide excision repair that is initiated upon stalling of RNA polymerase II (Pol II) on lesions in the template strand (3,4). DSBs tend to occur in transcriptionally active DNA (5,6) and, in recent years, it has become clear that HR is the preferred DSB repair pathway to deal with these transcription-disrupting lesions (7–10). The mechanism and regulation of this pathway, termed transcription-coupled HR, seems to be different from classical HR in S/G2 phase, although precise details of this pathway are not yet fully elucidated (11,12).

In response to a DSB in or near an actively transcribed gene, transcription is locally silenced. This involves the activity of DNA damage signaling factors including PARP1, chromatin remodeling by the NuRD and PBAF complexes, and WWP2-mediated degradation of Pol II (13–19). As a consequence of this transcription repression, nascent mRNA hybridizes to single-stranded template DNA leading to the formation of DNA-RNA hybrid structures called R-loops that are thought to be important for the recruitment of different DNA repair proteins but also need to be regulated themselves to prevent unwanted interference with DNA repair processes (20). In particular, R-loop-dependent RAD52 recruitment has been shown to facilitate HR in transcribed genes by promoting RAD51 nucleoprotein filament assembly (8,21). Also, RAD52 stimulates the recruitment of the endonuclease XPG. In addition to other factors such as RNaseH1 and SETX, XPG has been implicated in the processing of DSB-induced R-loops to allow proper DSB repair (8,22–24). However, many details of how R-loop processing is regulated and how this is coupled to transcriptional silencing and recruitment of DNA repair proteins to lesions are still unknown.

Chromatin modifications and structural rearrangements play important roles in regulating transcription and DNA repair. SWI/SNF is a family of heterogeneous ATP-dependent chromatin remodeling complexes that hydrolyze ATP to adjust chromatin conformation by sliding nucleosomes along DNA and evicting histones from chromatin (25). BRG1/SMARCA4 and BRM/SMARCA2 are the mutually exclusive core ATPases within the SWI/SNF family. Three major types of complexes are called BAF, PBAF and ncBAF, which share multiple core subunits including either BRG1 or BRM as catalytic subunit, but are each characterized by specific regulatory subunits (26). BAF complexes comprise either one of the two mutually exclusive subunits ARID1A/BAF250A or ARID1B/BAF250B. PBAF complexes contain complex-specific subunits such as ARID2 and PBRM1. ncBAF complexes contain the specific subunit BRD9. Mutations in SWI/SNF subunits are found in over 20% of human cancers, with ARID1A being the most frequently mutated subunit (26–28). SWI/SNF complexes have previously been implicated in the DNA damage response (DDR) (29–31), but the specific role of each different SWI/SNF complex and the way they may act together to promote DNA repair is still not understood.

In a *Caenorhabditis elegans* genetic screen, we found that SWI/SNF deficiency leads to increased UV-induced DNA damage sensitivity (32). Functional follow-up analysis in mammalian cells showed that both BRM and BRG1 regulate expression of the TFIIH subunit GTF2H1/p62, thus promoting the nucleotide excision repair pathway (33). Additionally, SWI/SNF complexes have been implicated in DSB repair, promoting both NHEJ and HR (29,34–40), but the various activities reported have made it difficult to deduce a unifying model of their activity (29). Also, PBAF subunits BRG1, PBRM1 and ARID2 were shown to mediate transcriptional silencing at DSB sites (13,14,30). Here, we uncover novel roles of ARID1A, ARID1B, BRG1 and BRM in transcription-coupled DSB repair. Our results suggest that different SWI/SNF complexes act coordinately to silence transcription, by promoting RPB1 eviction, and to facilitate R-loop resolution and DNA repair, by recruiting RNaseH1 and RAD52 to allow RAD51 loading to the chromatin.

## MATERIALS AND METHODS

### Cell lines, culture conditions and treatments

Cells used in this study are listed in Supplementary Table S1. U2OS, MRC-5 and HCT116 cells were cultured at 37°C in a humidified atmosphere with 5% CO_2_ in a 1:1 mixture of DMEM (Lonza) and Ham's F10 (Lonza) supplemented with 10% fetal calf serum and 1% penicillin-streptomycin. ARID1A-mAID-mClover, ARID1B-mAID-mClover and BRG1-mAID-mClover knock-in (KI) cells were generated by transiently transfecting osTIR1-expressing HCT116 cells (41) with a pLentiCRISPR-V2 plasmid encoding Cas9 and sgRNAs targeting ARID1A (TGGCCAGTCATGACAGCCGT), ARID1B (CAGTTATGACATAAGTGAGA) or BRG1 (GGGTCGAGACTGGAATGTCG) and with homology-directed repair templates containing the mAID-mClover-NeoR cassette from plasmid pMK289 and mAID-mClover-HygroR cassette from pMK290 (a gift from Masato Kanemaki (41)) flanked by 130–175 bp homology arms. Subsequently, cells were cultured in presence of 100 μg/ml hygromycin and 700 μg/ml neomycin for two weeks to select for successful recombination. HCT116 OsTIR1 stably expressing GFP-RAD52 were generated by transfecting pEGFP-C1-RAD52 (a kind gift of Kiyoshi Miyagawa (8)) and selected with G418 and FACS. U2OS cells stably expressing GFP-RAD52 and the fluorescent Cdt1-cell cycle marker were generated by transfection of an sgRNA targeting AAVS1 (GGGGCCACTAGGGACAGGAT), a homology-directed repair template containing GFP-RAD52 and a Blasticidin selection cassette flanked by 200 bp homology arms, and hCdt1-mKO2 (a kind gift of Bert van der Horst (42)). Cells were selected by FACS and blasticidin selection. For all cell lines, single-cell clones were isolated and verified by genotyping and by immunoblot. Plasmid transfections were performed using JetPei (Promega), according to the manufacturer's instructions. siRNA transfections were carried out 48 h before each experiment using Lipofectamine RNAiMax (Invitrogen) according to the manufacturer's protocol. siRNAs were purchased from Dharmacon and are listed on Supplementary Table S2. siRNAs efficiency was tested for each experiment by immunoblot (Supplementary Figure S7). For live cell imaging studies, cells were pre-treated for 1–2 h with inhibitors as indicated for each experiment. For inducing expression of GFP-RNaseH1(D210N), cells were incubated with 100 ng/ml doxycycline (Sigma) for 12 h. All chemicals, inhibitors and concentrations used are listed in Supplementary Table S3.

### Colony survival assay

For colony survival assays, cells were incubated for one day (with IR and cisplatin) or two days (with PARPi) in absence or presence of 40 ng/ml doxycycline and 100 nM auxin (3-indoleacetic acid, Sigma). Cells were then seeded in triplicate in six-well plates (700 cells/well) and immediately (for PARPi) or the next day (for IR and cisplatin) treated with increasing doses of the DNA damaging agent. After approximately seven days, colonies were fixed and stained. Fixing and staining solution: 0.1% w/v Coomassie Blue (Bio-Rad) was dispersed in a 50% Methanol, 10% Acetic Acid solution. Colonies were counted with the integrated colony counter GelCount (Oxford Optronix).

### DR-GFP assay and cell cycle profiling

HR efficiency was measured in U2OS cells with a stably integrated transgenic DR-GFP reporter (43), as previously described (44). Cells were treated with siRNAs and subsequently transfected with I-SceI-expression vector pCBASce (a gift from Maria Jasin; Addgene plasmid #26477) (45). 48 h after transfection, GFP-positive cells were assayed by flow cytometry. For cell cycle profiling, U2OS cells containing the DR-GFP reporter system were transfected with siRNAs, after 48 h transfected with pCBASce and 24 h later stained with propidium iodide. Cells were subjected to flow cytometry analysis on a BD LSRFortessaTM flow cytometer (BD Bioscience) using FACSDiva software. The percentage of cells in G1, S and G2/M phase was determined Flowing software 2.5.1 (by Perttu Terho in collaboration with Turku Bioimaging).

### Multiphoton laser microirradiation

Multiphoton laser microirradiation was performed using a Leica SP5 confocal microscope equipped with an environmental chamber set to 37°C and 5% CO2 as described (46,47). DSB-containing tracks (1- or 1.5 μm width) were generated with a Mira modelocked Ti:Sapphire laser (λ = 800 nm, pulselength = 200 fs, repetition rate = 76 MHz, and output power = 80 mW). For live cell imaging, confocal images were recorded before and after laser irradiation as indicated for each experiment. Data collection and analysis was performed using LAS X software (Leica). For immunofluorescence or transcription measurements, cells were microirradiated for 10 min, during which cells in ten consecutive fields of view were irradiated. Immediately or following a recovery period, as indicated per experiment, cells were fixed in 4% formaldehyde in PBS or ice-cold MeOH.

### Immunofluorescence

For immunofluorescence, cells were grown on coverslips and fixed with 4% formaldehyde in PBS. For BrdU detection, cells were pre-labelled with 30 μM 5-bromo-2′-deoxyuridine (BrdU; Sigma) for 48 h and incubated for 3 h with neocarzinostatin (Merck Millipore, N9162). For BrdU, RPA and RAD51 detection, 1 min pre-extraction with 0.5% Triton X-100 in CSK buffer (20 mM Hepes pH 7.6, 50 mM NaCl, 6 mM MgCl_2_, 300 mM sucrose) was performed prior to fixation. After this, cells were shortly permeabilized with 0.1% triton X-100, followed by incubation in blocking buffer (PBS containing 0.5% BSA and 0.15% glycine). Cells were incubated with primary antibodies (listed in Supplementary table S4) diluted in blocking buffer for 2 h at room temperature. Subsequently, cells were incubated with secondary antibodies (listed in Supplementary Table S5) for 1 h at room temperature. Cells were thoroughly washed in blocking buffer in between each step. DNA was stained using DAPI (Sigma) and slides were mounted using Aqua-Poly Mount (Polysciences, Inc). Images were acquired using an LSM700 confocal microscope (Carl Zeiss Micro Imaging Inc.).

### Transcription and R-loop measurements

To measure transcription activity at DSB sites, cells were incubated with 0.4 mM 5-ethynyl uridine (EU; Axxora) for 30 min, directly or 1 h after microirradiation. Cells were fixed with 4% formaldehyde in PBS for 15 min and permeabilized with 0.1% of Triton X-100. To visualize EU incorporation, cells were incubated in Click-it buffer containing 60 μM Atto 594 Azide (Atto Tec.), 50 mM Tris–HCl (pH 7.6), 4 mM CuSO_4_·5H_2_O (Sigma) and 10 mM ascorbic acid (Sigma) for 1 h and then washed with PBS containing 0.1% Triton X-100. After the Click-it reaction, immunofluorescence was performed to visualize sites of damage by immunostaining for γH2AX. To measure R-loops, cells were fixed using ice-cold MeOH for 10 min followed by 1 min permeabilization with ice-cold acetone. Cells were washed 3× in 4× SSC buffer and were blocked in 3% BSA in 0.1% Tween-20/SSC 4× for 1 h. Then, cells were incubated with S9.6 antibody at 1:1000 dilution. Secondary antibody anti-mouse Alexa Fluor® 594 (1:1000) was used and DNA was stained using 2 μg/μl of DAPI. Coverslips were mounted using Aqua-Poly Mount (Polysciences, Inc.). Samples were stored in dark at 4°C prior to imaging.

### Immunoblotting

To detect proteins by immunoblot, cells were washed with PBS, lysed in sample buffer (0.125 M Tris–HCl pH 6.8, 2% SDS, 0.005% bromophenol blue, 21% glycerol, 4% β-mercaptoethanol) and boiled for 5 min at 98°C. Equal amounts of proteins were separated on 4–12% SDS-PAGE gels (Invitrogen) and transferred onto PDVF membranes (0.45 μm, Merck Millipore) at 4°C for 15 h at 30 V in transfer buffer (25 mM Tris, 190 mM Glycine, 10% MeOH). Membranes were blocked with 5% BSA in PBS and probed with primary antibodies for 2 h at room temperature or overnight at 4°C. Membranes were washed with PBS-Tween (0.05%) and incubated with secondary antibodies coupled to IRDye (LI-COR) for 1 h to visualize proteins using an Odyssey CLx Infrared Imaging System (LI-COR Biosciences) and Image Studio Lite software v5.2 (LI-COR Biosciences). Antibodies are listed in Supplementary Tables S4 and S5. BAF47 antibody was kindly provided by Jan van der Knaap (48).

### S9.6 antibody purification

For R-loop detection, the RNA:DNA specific S9.6 antibody was purified from the S9.6 producing hybridoma mouse cell line purchased from ATCC (HB-8730). Hybridoma cells were initially cultured in DMEM (Lonza), containing 10% Fetal Bovine serum, as recommended by ATCC. Following the initial establishment period, cells were adapted to PFHM-II serum free growth medium suitable for MAb production (Gibco, Life Sciences cat. 12040-077), as recommended by the manufacturer. Briefly, cells at the log phase of growth (1 × 10^6^ cells/ml) were sub-cultured in gradually increasing ratio of PFHM-II to DMEM, at density of 2 × 10^5^ cells/ml, until they were able to sustain consistent growth and viability in 100% complete serum-free PFHM-II. Cell viability was determined at each sub-cultivation by trypan-blue exclusion. S9.6 monoclonal antibodies (mouse IgGs) were purified from 0.45 micron-filtered hybridoma supernatant, by column chromatography, using a HiTrap™ MabSelect SuRe™ column (GE Healthcare, cat. 29-0491-04), on an AKTA START protein purification system equipped with an automated fraction collector (Cytiva), as recommended by the manufacturers. Eluted antibody purity was verified by SDS-PAGE followed by Collodia Coomassie blue R-250 staining, and concentration was determined using by the BCA protein assay (Pierce, ThermoScientific, cat.23225).

### mClover immunoprecipitation and SILAC-based proteomics

For immunoprecipitation of ARID1B-mAID-mClover and BRG1-mAID-mClover complexes, whole cell lysate of normally cultured cells was used. For immunoprecipitation of ARID1A-mAID-mClover, stable isotope labeling of amino acids in culture (SILAC) was used, for which cells were grown in DMEM containing 10% dialyzed FBS (Gibco), 10% GlutaMAX (Life Technologies), penicillin/streptomycin (Life Technologies), unlabeled l-arginine–HCl and l-lysine–HCl or 13C6,15N4l-arginine–HCl and 13C6,15N2l-lysine–2HCl (Cambridge Isotope Laboratories), respectively. To lyse cells, cells were trypsinized and sonicated in IP buffer (30 mM HEPES buffer pH 7.5, 130 mM NaCl, 1 mM MgCl_2_, 0,5% Triton X-100) containing EDTA-free protease inhibitors (Roche), followed by benzonase (Millipore) incubation. Equal amounts of protein extracts were incubated with GFP-Trap®_A beads (Chromotek), and extensively washed. Bound proteins were eluted by boiling of the beads in Laemmli-SDS sample buffer and separated in SDS-PAGE gels. ARID1B-mAID-mClover and BRG1-mAID-mClover pulldowns were visualized by immunoblotting. ARID1A-mAID-mClover pulldown bands were visualized with Coomassie (SimplyBlue; Invitrogen). Subsequently, the SDS-PAGE gel lanes were cut into 2-mm slices and subjected to in-gel reduction with dithiothreitol, alkylation with iodoacetamide (98%; D4, Cambridge Isotope Laboratories) and digestion with trypsin (sequencing grade; Promega). Nanoflow liquid chromatography tandem mass spectrometry (nLC-MS/MS) was performed on an EASY-nLC coupled to an Orbitrap Fusion mass spectrometer (ThermoFisher Scientific), operating in positive ion mode. Peptide mixtures were trapped on a ReproSil C18 reversed phase column (Dr Maisch; 1.5 cm × 100 μm) at a rate of 8 μl/min. Peptides were separated on a ReproSil-C18 reversed-phase column (Dr Maisch; 15 cm × 50 μm) using a linear gradient of 0–80% acetonitrile (in 0.1% formic acid) for 170 min at a rate of 200 nl/min. The elution was directly sprayed into the electrospray ionization source of the mass spectrometer. Spectra were acquired in continuum mode; fragmentation of the peptides was performed in data-dependent mode. Raw mass spectrometry data were analyzed using the MaxQuant software suite (version 2.0.3.0). A false discovery rate of 0.01 for proteins and peptides and a minimum peptide length of seven amino acids were set. The Andromeda search engine was used to search the MS/MS spectra against the Uniprot database (taxonomy: Homo sapiens, release 2021). A maximum of three missed cleavages was allowed. The enzyme specificity was set to ‘trypsin’, and cysteine carbamidomethylation was set as a fixed modification. SILAC protein ratios were calculated as the median of all peptide ratios assigned to the protein. Before further statistical analysis, known contaminants and reverse hits were removed.

### Statistical analysis

Mean values and SEM error bars are shown for each experiment. Where indicated, unpaired two-tailed t- or unpaired one-way ANOVA tests were used to determine statistical significance between groups. All analysis were performed in Graph Pad Prism version 8.3.0 for Windows (GraphPad Software, La Jolla California USA). P values are indicated as number in each figure.

## RESULTS

### SWI/SNF complexes promote DSB repair

**Figure 1.**
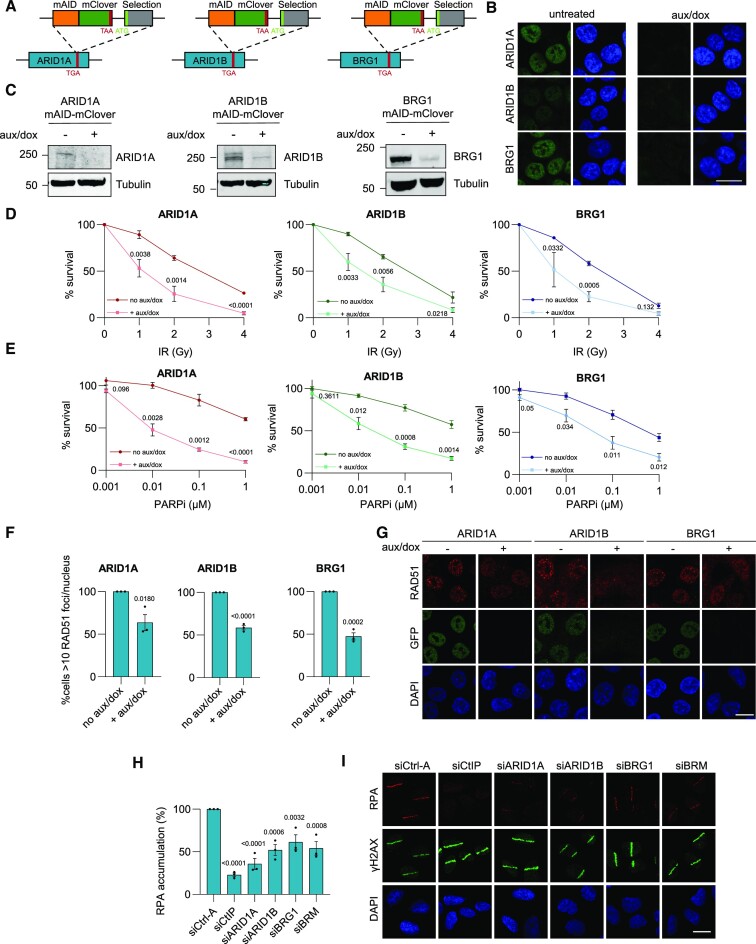
Different SWI/SNF complexes promote homologous recombination. (**A**) Schematic of the C-terminal tagging of endogenous ARID1A, ARID1B and BRG1 with mAID-mClover. Each homology-directed repair template also contained a neomycin or hygromycin gene for selection of transgenic cells. (**B**) Representative images showing nuclear localization of mAID-mClover-tagged ARID1A, ARID1B and BRG1 in fixed HCT116 cells. Cells were untreated or incubated with 0.1 μM auxin and 40 ng/ml doxycycline (aux/dox) for 48 h. DNA is stained with DAPI. (**C**) Immunoblot analysis of lysate of ARID1A-, ARID1B-, BRG1-mAID-mClover knock-in HCT116 cells untreated or treated with 0.1 μM auxin and 40 ng/ml doxycycline (Aux/Dox) for 48 h. Blots were stained with the indicated antibodies and tubulin was used as loading control. (**D**) Ionizing radiation (IR) colony survival assay of ARID1A-, ARID1B- and BRG1-mAID-mClover knock-in HCT116 cells incubated with or without auxin and doxycycline (aux/dox). Mean and SEM of three independent experiments. (**E**) PARPi colony survival assay of ARID1A-, ARID1B- and BRG1-mAID-mClover knock-in HCT116 cells incubated with or without auxin and doxycycline (aux/dox). Mean and SEM of three independent experiments. (**F**) Quantification of the percentage of ARID1A-, ARID1B-, BRG1-mClover-mAID HCT116 knock-in cells with RAD51 foci that had more than 10 foci/nucleus. HCT116 cells were incubated with or without auxin and doxycycline (aux/dox) for 48 h before irradiation. Cells were treated with 4 Gy ionizing radiation and fixed after 2 h. Mean and SEM of three independent experiments. (**G**) Immunofluorescence images showing RAD51 foci in irradiated ARID1A-, ARID1B- and BRG1-mAID-mClover knock-in HCT116 cells incubated with or without auxin and doxycycline (aux/dox) as described in (**F**). Cells were stained with RAD51 and GFP (to visualize mClover) antibodies and DNA was stained with DAPI. (**H**) Relative quantification of the intensity of RPA staining in laser-induced DNA damage tracks 30 min after multiphoton microirradiation of U2OS cells transfected with the indicated siRNAs. Mean and SEM of three independent experiments. **(I**) Immunofluorescence images showing RPA and γH2AX localization to DNA damage 30 min after multiphoton microirradiation of U2OS cells transfected with the indicated siRNAs. Cells were stained with antibodies against RPA34 and γH2AX. DNA is stained with DAPI. In each graph, numbers indicate p values, which were obtained using an unpaired *t*-test (in D–F) or one-way ANOVA test (in H). Scale bar, 10 μm.

To explore the function of different SWI/SNF complexes in the DDR, we knocked in a mini-Auxin-Inducible-Degron (mAID) tag fused to mClover at the endogenous locus of SWI/SNF subunits ARID1A, ARID1B and BRG1, using CRISPR/Cas9 in HCT116 cells stably expressing doxycycline-inducible OsTIR1 (Figure 1A) (41). C-terminally tagged endogenous ARID1A, ARID1B and BRG1 were exclusively localized in the nucleus (Figure 1B), in line with their nuclear function. Quantification of the mClover intensities showed that ARID1A and BRG1 expression is more than two times higher than that of ARID1B (Supplementary Figure S1A). Efficient depletion of fluorescent ARID1A, ARID1B and BRG1 was achieved by incubation with doxycycline and auxin, which respectively induce expression and activation of OsTIR1 that forms a functional Skp1-Cullin-F-box ubiquitin ligase complex targeting the mAID tag, allowing degradation of mAID-tagged proteins (Figure 1B and C). We confirmed by quantitative proteomics of immunoprecipitated ARID1A-mAID-mClover that endogenously-tagged ARID1A was normally incorporated into the BAF complex (Supplementary Figure S1B and C). Similarly, we confirmed by immunoprecipitation of BRG1-mAID-mClover (Supplementary Figure S1D) and ARID1B-mAID-mClover (Supplementary Figure S1E) that these endogenously-tagged factors interact with other relevant SWI/SNF complex subunits. These results indicate that the mClover tag does not interfere with formation of SWI/SNF complexes.

To investigate if SWI/SNF complexes function in DSB repair, we performed clonogenic survival assays using various DNA damaging agents. Depletion of SWI/SNF subunits with the mAID degron system clearly sensitized cells to ionizing radiation (IR) treatment (Figure 1D). We also tested sensitivity to cisplatin, which generates interstrand crosslinks (ICLs) whose repair depends on HR (49,50), and to the PARP inhibitor KU0058948 (PARPi), to which HR-deficient cells are sensitive (51). In line with previous findings for ARID1A and BRG1 (34,35,52), cells lacking these factors are hypersensitive to both cisplatin and PARPi (Figure 1E and Supplementary Figure S2A), suggesting that these SWI/SNF factors are important for HR. Similarly, cells lacking ARID1B are sensitive to both treatments (Figure 1E and Supplementary Figure S2A), indicating that different SWI/SNF BAF complexes, i.e. formed by the mutually exclusive subunits ARID1A or ARID1B, all play a role in HR.

### SWI/SNF facilitates HR by promoting end resection and RAD51 accumulation

To confirm that different SWI/SNF complexes participate in HR, we depleted ARID1A, ARID1B, BRG1 and, in addition, BRM using siRNA in U2OS cells, and measured HR efficiency using the I-SceI DR-GFP assay (44). We used U2OS cells, as alternative to HCT116, to verify that the studied DNA repair role of SWI/SNF subunits is not cell-type specific. The DR-GFP assay measures HR-mediated restoration of a mutated GFP gene following DSB induction by I-SceI, using flow cytometry. Depletion of the different SWI/SNF subunits mildly reduced HR efficiency, as compared to depletion of control HR factor BRCA1, without strongly affecting cell cycle phase distribution (Supplementary Figure S2B, C). To corroborate these findings, we measured RAD51 foci formation after IR in our knock-in HCT116 cell lines and observed that this was impaired upon depletion of ARID1A, ARID1B and BRG1 (Figure 1F, G). These results suggest that different SWI/SNF complexes promote the loading of RAD51.

Given the fact that efficient RAD51 loading requires DNA end resection, we investigated if cells lacking SWI/SNF had resection defects. To this end, we labelled siRNA-transfected U2OS cells with 5-bromo-2′deoxyridine (BrdU) and treated these cells with the radiomimetic drug neocarzinostatin (NCS) to generate DSBs. Subsequently, we performed immunofluorescence with anti-BrdU antibodies under non-denaturing conditions to detect single stranded DNA (ssDNA) as direct measure of resected DNA (53). A decreased number of BrdU foci per cell confirmed that ARID1A, ARID1B, BRG1 and BRM depletion causes DNA end resection problems (Supplementary Figure S2D, E). To independently validate these findings, we measured recruitment of RPA to resected DNA at DSB sites. To this end, we microirradiated siRNA-transfected U2OS cells using 800 nm multiphoton laser to generate DSB tracks (46) and performed immunofluorescence to visualize RPA binding to ssDNA. In line with the decreased BrdU foci, depletion of ARID1A, ARID1B, BRG1 and BRM clearly reduced RPA accumulation at the site of damage, marked by γH2AX staining (Figure 1H, I). These results, therefore, indicate that multiple different SWI/SNF complexes, containing the mutually exclusive BRG1 or BRM ATPase and/or the ARID1A or the ARID1B regulatory subunit, promote HR by facilitating DNA end resection to allow RAD51 binding to DNA.

### PARP and HDAC-dependent DSB recruitment of different SWI/SNF complexes

**Figure 2.**
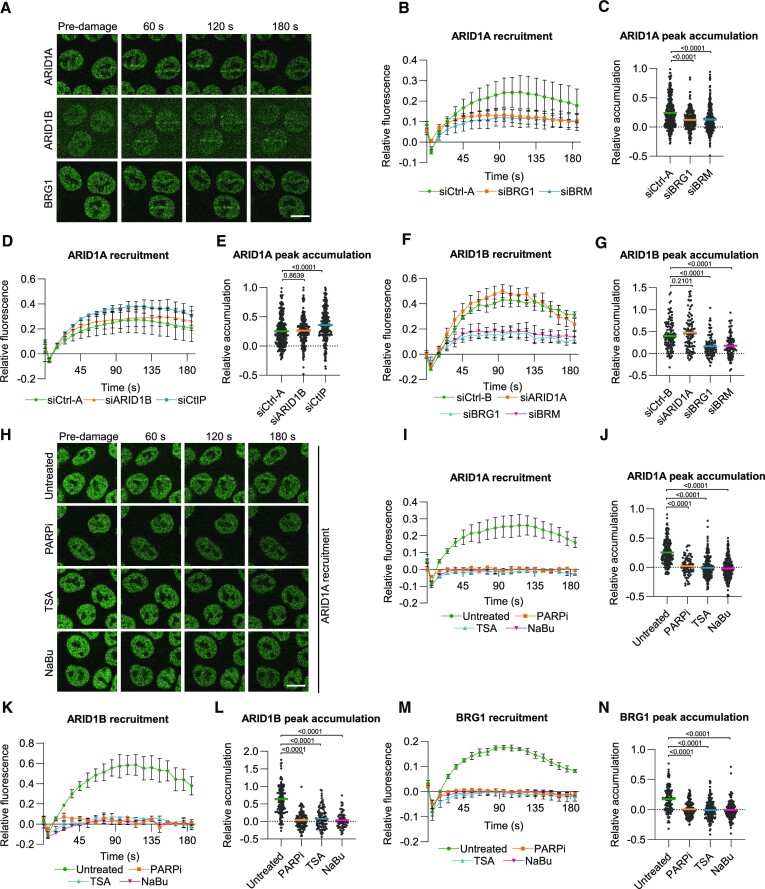
Different SWI/SNF complexes are recruited to double-strand breaks. (**A**) Representative images showing the real-time accumulation of endogenously mAID-mClover-tagged ARID1A, ARID1B and BRG1 in multiphoton laser-generated DNA damage tracks in HCT116 cells. (**B**) Quantification of real-time ARID1A-mAID-mClover recruitment to laser- tracks in HCT116 cells treated with control, BRG1 or BRM siRNA. Mean and SEM of four independent experiments. (**C**) Quantification of peak accumulation (85–105 s) in experiments shown in (B). Cells were pooled and for siCtrl-A *n* = 360, siBRG1 *n* = 306 and siBRM *n* = 345. (**D**) Quantification of real-time ARID1A-mAID-mClover recruitment to laser-tracks in HCT116 cells treated with control, ARID1B and CtIP siRNA. Mean and SEM of three independent experiments. (**E**) Quantification of peak accumulation (85–105 s) in experiments shown in (D). Cells were pooled and for siCtrl-A *n* = 308, siARID1B *n* = 255 and siCtIP *n* = 292. (**F**) Quantification of real-time ARID1B-mAID-mClover recruitment to laser-tracks in HCT116 cells treated with control, siARID1A, BRG1 or BRM siRNA. Mean and SEM of three independent experiments. (**G**) Quantification of peak accumulation (85–105 s) in experiments shown in (F). Cells were pooled and for siCtrl-B *n* = 118, siARID1A *n* = 102, siBRG1 *n* = 98 and siBRM *n* = 95. (**H**) Representative images showing the real-time accumulation of endogenously mClover-tagged ARID1A in HCT116 cells untreated or treated with 10 μM PARPi (KU0058948), 1 μM TSA or 5 mM NaBu. (**I**) Quantification of real-time ARID1A-mAID-mClover recruitment to laser-tracks in HCT116 cells untreated or treated with 10 μM PARPi (KU0058948), 1 μM TSA or 5 mM NaBu. Mean and SEM of three independent experiments (**J**) Quantification of peak accumulation (85–105 s) in experiments shown in (I). Cells were pooled and for untreated *n* = 217 cells, PARPi *n* = 76 cells, TSA *n* = 220 cells and NaBu *n* = 226 cells. (**K**) Quantification of real-time ARID1B-mAID-mClover recruitment to laser-tracks in HCT116 cells untreated or treated with 10 μM PARPi (KU0058948), 1 μM TSA or 5 mM NaBu. Mean and SEM of three (PARPi, TDA) or two (NaBu) independent experiments. (**L**) Quantification of peak accumulation (85–105 s) in experiments shown in (K). Cells were pooled and for untreated *n* = 146 cells, PARPi *n* = 107 cells, TSA *n* = 98 cells and NaBu *n* = 68 cells. (**M**) Quantification of real-time BRG1-mAID-mClover recruitment to laser-tracks in HCT116 cells untreated or treated with 10 μM PARPi (KU0058948), 1 μM TSA or 5 mM NaBu. Mean and SEM of three independent experiments. (**N**) Quantification of peak accumulation (85–105 s) in experiments shown in (M). Cells were pooled and for untreated *n* = 133 cells, PARPi *n* = 160 cells, TSA *n* = 172 cells and NaBu *n* = 144 cells. For quantification of the DNA damage recruitment, the relative fluorescence, corrected for background signal, was measured over time in the DNA damage tracks and normalized to the pre-damage fluorescence intensity. In each graph, numbers indicate *P* values obtained using a one-way ANOVA test. Scale bar, 10 μm.

To dissect how SWI/SNF complexes participate in HR, we analyzed the real-time DNA damage recruitment of mClover-tagged ARID1A, ARID1B and BRG1 at DSB tracks generated by multiphoton laser. This showed that all three subunits were rapidly recruited to the damaged area, in all cells tested (Figure 2A). This laser-induced DNA damage recruitment is in line with DSB recruitment previously observed for ARID1A, ARID1B and BRG1 using various methods (15,34–36).

We depleted ARID1A, ARID1B, BRG1 and BRM to study their interdependent recruitment to DSBs. While ARID1A and ARID1B recruitment was completely independent of each other, accumulation of both was partially reduced when BRG1 or BRM were depleted (Figure 2B–G). These data confirm that ARID1A and ARID1B are mutually exclusive with each other in SWI/SNF and suggest that both are recruited to DSBs as part of two different types of SWI/SNF BAF complexes, i.e. one containing BRG1 and the other containing BRM. Moreover, this finding indicates that BRM-containing SWI/SNF complexes are also recruited to DSBs. We could not test this directly, as we did not succeed in generating stable mClover-BRM knock-in cells. However, ectopically expressed GFP-tagged BRM has previously been shown to localize to laser-induced DNA damage (15,54), confirming our results. These results show that at least four different SWI/SNF complexes are recruited to DSBs and therefore indicate that multiple different SWI/SNF subunits act in HR.

We tested whether siRNA-mediated depletion or chemical inhibition of different factors implicated in DSB repair affected ARID1A recruitment. In line with a role upstream of DNA end resection, we found that ARID1A recruitment was elevated upon depletion of the DNA end resection factor CtIP (Figure 2D and E). Furthermore, ARID1A accumulation at DSBs seemed independent of DNA damage signaling via ATM or ATR, as evaluated with inhibitors against both kinases (Supplementary Figure S3A and B), and of later HR factors BRCA1 and BRCA2, as evaluated with siRNA (Supplementary Figure S3C and D). Strikingly, however, inhibition of PARP activity by PARPi completely abolished ARID1A recruitment to DNA damage (Figure 2H–J). We then found that PARPi also clearly inhibited ARID1B and BRG1 recruitment to DSBs (Figure 2K–N). These results indicate that PARylation regulates the recruitment of different SWI/SNF complexes to DSBs, as has also previously been observed for other ATP-dependent chromatin remodelers such as CHD4 (55,56), CHD2 (57) and CHD7 (58).

Some chromatin remodelers, such as CHD4 (56), are in complex with histone deacetylases (HDACs) that modify acetylation histone marks after DNA damage. Therefore, we tested whether SWI/SNF recruitment was affected by HDAC inhibition using trichostatin A (TSA) and sodium butyrate (NaBu). Surprisingly, recruitment of ARID1A was completely suppressed by both TSA and NaBu treatment, suggesting that histone deacetylation is needed for ARID1A accumulation at DNA damage (Figure 2H–J). To corroborate this result, we inhibited histone acetylation using the p300 histone acetyltransferase inhibitor CTK7A and observed that this significantly increased ARID1A accumulation (Supplementary Figure S3E and F). Moreover, we observed that ARID1B and BRG1 recruitment was abolished upon treatment with either TSA or NaBu (Figure 2K–N). Taken together, these results indicate that different SWI/SNF complexes are recruited to DSBs in a manner dependent on PARylation and histone deacetylation.

### NuRD and transcription-dependent DSB recruitment of different SWI/SNF complexes

**Figure 3.**
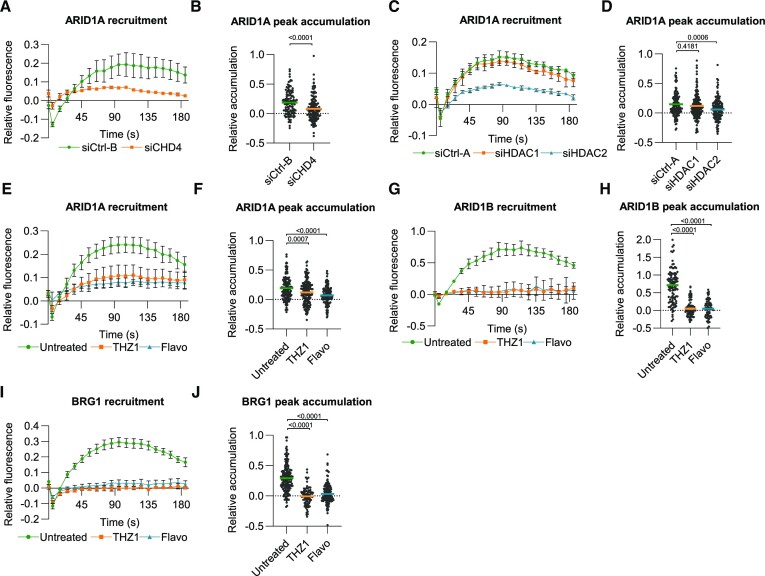
SWI/SNF localizes to double-strand breaks in transcriptionally active DNA. (**A**) Quantification of real-time ARID1A-mAID-mClover recruitment to laser- tracks in HCT116 cells treated with control and CHD4 siRNA. Mean and SEM of three independent experiments. (**B**) Quantification of peak accumulation (85–105 s) in experiments shown in (A). Cells were pooled and for siCtrl-B *n* = 121 and siCHD4 *n* = 140. (**C**) Quantification of real-time ARID1A-mAID-mClover recruitment to laser-tracks in HCT116 cells treated with control, HDAC1 and HDAC2 siRNA. Mean and SEM of three independent experiments. (**D**) Quantification of peak accumulation (85–105 s) in experiments shown in (C). Cells were pooled and siCtrl-A *n* = 141, siHDAC1 *n* = 162 and siHDAC2 *n* = 152. (**E**) Quantification of real-time ARID1A-mAID-mClover recruitment to laser-tracks in HCT116 cells untreated or treated with 1 μM THZ1 or flavopiridol (Flavo). Mean and SEM of three independent experiments. (**F**) Quantification of peak accumulation (85–105 s) in experiments shown in (E). Cells were pooled and for untreated *n* = 142, THZ1 *n* = 172 and flavopiridol *n* = 154. (**G**) Quantification of real-time ARID1B-mAID-mClover recruitment to laser-tracks in HCT116 cells untreated or treated with 1 μM THZ1 or flavopiridol. Mean and SEM of three (THZ1) or two (flavopiridol) independent experiments. (**H**) Quantification of peak accumulation (85–105 s) in experiments shown in (G). Cells were pooled and for untreated *n* = 114, THZ1 *n* = 78 and flavopiridol *n* = 66. (**I**) Quantification of real-time BRG1-mAID-mClover recruitment to laser-tracks in HCT116 cells untreated or treated with 1 μM THZ1 or flavopiridol. Mean and SEM of three independent experiments. **(J)** Quantification of peak accumulation (85–105 s) in experiments shown in (I). Cells were pooled and for untreated *n* = 166, THZ1 *n* = 82 and Flavopiridol *n* = 143. For quantification of the DNA damage recruitment, the relative fluorescence, corrected for background signal, was measured over time in the DNA damage tracks and normalized to the pre-damage fluorescence intensity. In each graph, numbers indicate *P* values obtained using an unpaired *t*-test unpaired *t*-test (in B) or one-way ANOVA test (in D, F, H, J).

CHD4 is the core catalytic subunit of the NuRD complex family of chromatin remodelers that also contain HDAC1 and/or HDAC2 (59). As CHD4 and HDAC1 are recruited to laser-induced DNA damage in a PARP-dependent manner (55,56,58), we wondered whether the observed HDAC-dependent recruitment of SWI/SNF factors is due to involvement of the NuRD complex. To test this, we depleted CHD4, HDAC1 and HDAC2 by siRNA and tested ARID1A recruitment in our knock-in HCT116 cells. Noticeably, we observed that ARID1A accumulation was reduced upon depletion of CHD4 and HDAC2 but not of HDAC1 (Figure 3A–D). Depletion of HDAC3, another exclusively nuclear class I HDAC like HDAC1 and HDAC2, also did not affect ARID1A recruitment (Supplementary Figure S3G and H). These results suggest that ARID1A recruitment to DSBs depends on the activity of a NuRD complex containing both CHD4 and HDAC2.

The NuRD complex has previously been implicated in a transcription-coupled DDR pathway that promotes HR, as CHD4 accumulation to DNA damage was found to be dependent on transcription (15). To test whether recruitment of the different SWI/SNF subunits is also transcription dependent, we treated the knock-in cells with two different transcription elongation inhibitors, i.e. the CDK7-inhibitor THZ1 (60) and flavopiridol (61). Strikingly, we observed that ARID1A recruitment was partially reduced and that ARID1B and BRG1 recruitment was completely abolished (Figure 3E–J). These results indicate that the different SWI/SNF complexes play a role in HR in transcriptionally active genes.

### ARID1A promotes RAD52 recruitment to DSBs

**Figure 4.**
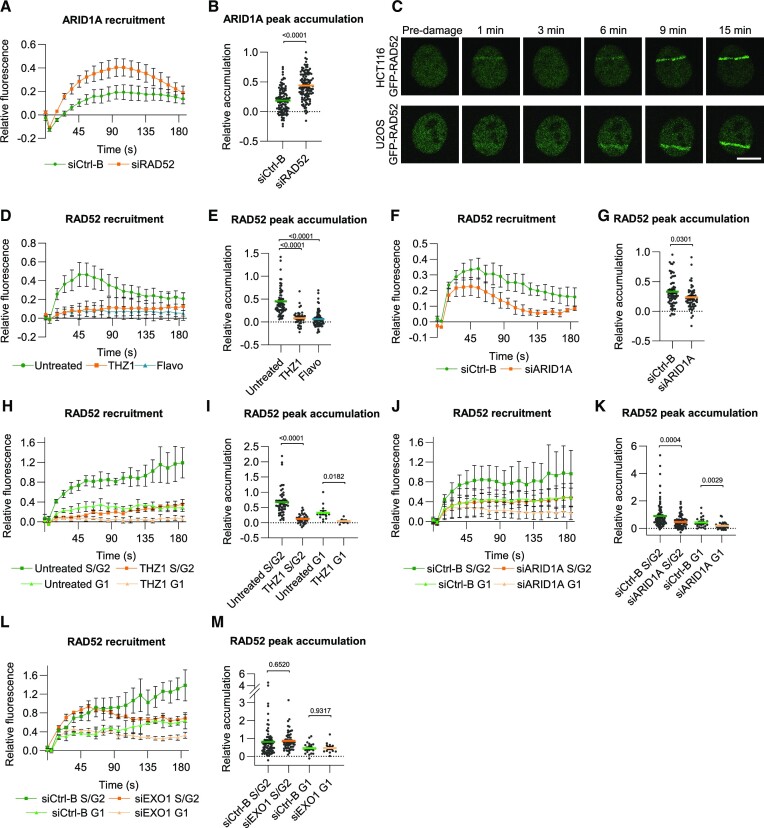
SWI/SNF promotes RAD52 recruitment to DSBs. (**A**) Quantification of real-time ARID1A-mAID-mClover recruitment to laser- tracks in HCT116 cells treated with control and RAD52 siRNA. Mean and SEM of three independent experiments. (**B**) Quantification of peak accumulation (85–105 s) in experiments shown in (A). Cells were pooled and for siCtrl-B *n* = 132 and siRAD52 *n* = 119. (**C**) Representative images showing the real-time accumulation of GFP-RAD52 to laser-tracks in HCT116 and in U2OS cells. (**D**) Quantification of real-time GFP-RAD52 recruitment to laser-tracks in HCT116 cells untreated or treated with 1 μM THZ1 or 1 μM flavopiridol (Flavo). Mean and SEM of three (Flavo) or two (THZ1) independent experiments (**E**). Quantification of peak accumulation (35–55 s) in experiments shown in (D). Cells were pooled and for untreated *n* = 71, THZ1 *n* = 42 and Flavopiridol *n* = 85. (**F**) Quantification of real-time GFP-RAD52 recruitment to laser-tracks in HCT116 cells treated with control or ARID1A siRNA. Mean and SEM of three independent experiments. (**G**) Quantification of peak accumulation (35–55 s) in experiments shown in (F). Cells were pooled and for siCtrl-B *n* = 69 and siARID1A *n* = 62. (**H**) Quantification of real-time GFP-RAD52 recruitment to laser-tracks in hCdt1-mKO2-transgenic U2OS cells untreated or treated with 1 μM THZ1. hCdt1-mKO2 expression was used as marker for G1 cell cycle phase. Mean and SEM of three independent experiments (**I**) Quantification of peak accumulation (45–65 s) in experiments shown in (H). Cells were pooled and for untreated G1 *n* = 14, untreated S/G2 *n* = 53, THZ1 S/G2 *n* = 36 and THZ1 G1 *n* = 6. (**J**) Quantification of real-time GFP-RAD52 recruitment to laser-tracks in hCdt1-mKO2-transgenic U2OS cells treated with control or ARID1A siRNA. hCdt1-mKO2 expression was used as marker for G1 cell cycle phase. Mean and SEM of five independent experiments. (**K**) Quantification of peak accumulation (45–65 s) in experiments shown in (J). Cells were pooled and for siCtrl-B S/G2 *n* = 68, siCtrl-B G1 *n* = 17, siARID1A G2/S *n* = 58 and siARID1A G1 *n* = 23. (**L**) Quantification of real-time GFP-RAD52 recruitment to laser tracks in hCdt1-mKO2-transgenic U2OS cells treated with control or EXO1 siRNA. hCdt1-mKO2 expression was used as marker for G1 cell cycle phase. Mean and SEM of three independent experiments. (**M**) Quantification of peak accumulation (45–65 s) in experiments shown in (L). Cells were pooled and for siCtrl-B S/G2 *n* = 67, siCtrl-B G1 *n* = 9, siEXO1 G2/S *n* = 58 and siEXO1 G1 *n* = 13. For quantification of the DNA damage recruitment, the relative fluorescence, corrected for background signal, was measured over time in the DNA damage tracks and normalized to the pre-damage fluorescence intensity. In each graph, numbers indicate p values obtained using an unpaired *t*-test (in B, G, I, K, M) or one-way ANOVA test (in E). Scale bar, 10 μm.

Transcription-coupled HR was proposed to involve transcription- and R-loop-dependent RAD52 recruitment to DSBs, to stimulate DNA end resection and RPA and RAD51 loading (7,8,11,12,21,22,62). Therefore, we depleted RAD52 and observed that ARID1A accumulation was increased (Figure 4A and B), indicating that ARID1A associates more with damaged chromatin and that RAD52 likely acts downstream of ARID1A. Thus, to test if ARID1A acts in an upstream step of RAD52 and promotes its recruitment to DSBs, we generated HCT116 cells stably expressing GFP-tagged RAD52 (Supplementary Figure S3I) and tested GFP-RAD52 recruitment to DSB laser tracks. Strikingly, we noticed that GFP-RAD52 was recruited to laser-induced DNA damage in a biphasic manner, in which a rapid and transient first wave of RAD52 accumulation (within ∼1 min) was followed by a slower, but more pronounced and persistent second wave of accumulation (Figure 4C). The first accumulation wave was completely transcription dependent (Figure 4D, E), as noted previously (8). Also, depletion of ARID1A by siRNA clearly reduced this first wave of RAD52 recruitment (Figure 4F, G), suggesting that transcription-dependent ARID1A/BAF complex activity acts upstream of and promotes this initial RAD52 recruitment.

**Figure 5.**
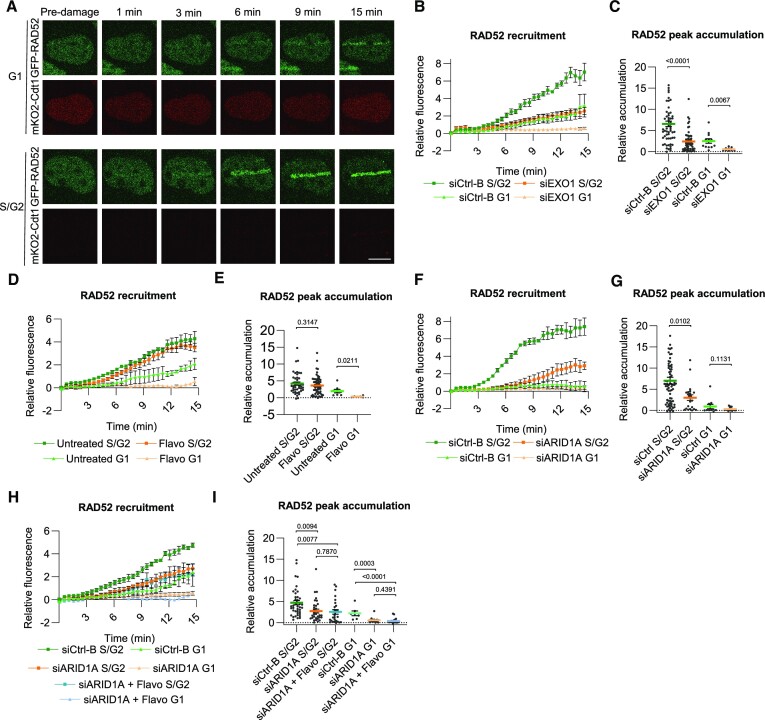
ARID1A and EXO1 promote stable RAD52 recruitment to DSBs. (**A**) Representative images showing the real-time accumulation of GFP-RAD52 to laser-tracks in hCdt1-mKO2-transgenic U2OS cells either in cell cycle phase G1 (hCdt1-mKO2 positive) or in S/G2 (hCdt1-mKO2 negative). (**B**) Quantification of real-time GFP-RAD52 recruitment to laser-tracks in hCdt1-mKO2-transgenic U2OS cells treated with control or EXO1 siRNA. Mean and SEM of three independent experiments. (**C**) Quantification of peak accumulation (815–875 s) in experiments shown in (B). Cells were pooled and for siCtrl-B S/G2 *n* = 59, siCtrl-B G1 *n* = 12, EXO1 S/G2 *n* = 52 and EXO1 G1 *n* = 9. (**D**) Quantification of real-time GFP-RAD52 recruitment to laser-tracks in hCdt1-mKO2-transgenic U2OS cells untreated or treated with 1 μM flavopiridol (Flavo). Mean and SEM of three independent experiments. (**E**) Quantification of peak accumulation (815–875 s) in experiments shown in (D). Cells were pooled and for untreated S/G2 *n* = 44, untreated G1 *n* = 8, flavopiridol s/G2 *n* = 57 and flavopiridol G1 *n* = 6. (**F**) Quantification of real-time GFP-RAD52 recruitment to laser-tracks in hCdt1-mKO2-transgenic U2OS cells treated with control or ARID1A siRNA. Mean and SEM of three independent experiments. (**G**) Quantification of peak accumulation (815–875 s) in experiments shown in (F). Cells were pooled and for siCtrl-B S/G2 *n* = 73, siCtrl-B G1 *n* = 11, siARID1A G2/S *n* = 22 and siARID1A G1 *n* = 22. (**H**) Quantification of real-time GFP-RAD52 recruitment to laser-tracks in hCdt1-mKO2-transgenic U2OS cells treated with control or ARID1A siRNA with or without 1 μM flavopiridol treatment. Mean and SEM of three independent experiments. (**I**) Quantification of peak accumulation (815–875 s) in experiments shown in (H). Cells were pooled and for siCtrl-B S/G2 *n* = 42, siCtrl-B G1 *n* = 7, siARID1A G2/S *n* = 34, siARID1A G1 *n* = 16, siARID1A + flavopiridol S/G2 *n* = 27 and siARID1A + flavopiridol G1 *n* = 19. hCdt1-mKO2 expression was used as marker for G1 cell cycle phase. For quantification of the DNA damage recruitment, the relative fluorescence, corrected for background signal, was measured over time in the DNA damage tracks and normalized to the pre-damage fluorescence intensity. In each graph, numbers indicate *P* values obtained using an unpaired *t*-test (in C, E, G, I). Scale bar, 10 μm.

To test if RAD52 recruitment depends on the cell cycle phase and to further characterize the second accumulation wave, we generated U2OS cells that stably express a human Cdt1 fragment fused to mOrange2 (hCdt1-mKO2) as live G1 cell cycle marker (Sakaue-Sawano *et al.* 2008) and used CRISPR/Cas9 to knock-in GFP-RAD52 cDNA in the AAVS1 locus (Smith *et al.* 2008) of these cells (Supplementary Figure S3J). We used U2OS cells as alternative to HCT116 to verify that observed phenotypes are not cell type specific. Using this cell line, we again found that RAD52 is recruited to DSBs in a biphasic manner (Figure 4C). Furthermore, recruitment was observed in all cell cycle phases, but was substantially lower in G1 compared to S/G2 phases (Figures 4H and 5A). However, in both S/G2 and G1 cell cycle phases, the first wave of RAD52 accumulation was strongly dependent on transcription and on ARID1A (Figure 4H–K), confirming the observations in HCT116 cells. We also tested if this first wave is dependent on extensive DNA end-resection, by depleting EXO1, but we did not observe that this affected the rapid initial recruitment of RAD52 to DSB laser tracks (Figure 4L, M).

In contrast, the second accumulation wave of RAD52 was strongly reduced after EXO1 depletion, in G1 and S/G2 cell cycle phases (Figure 5A–C). Also, we found that this second wave of accumulation in S/G2 phase was not significantly reduced upon transcription inhibition in S/G2 phase, but only in the G1 cell cycle phase (Figure 5D, E). Contrarily, depletion of ARID1A led to a strong reduction of the second RAD52 wave in S/G2 cells (Figure 5F, G). Because ARID1A recruitment itself is partially dependent on transcription (Figure 3E, F), we combined transcription inhibition with ARID1A depletion, but observed that this did not further reduce RAD52 recruitment (Figure 5H, I). Together, our results suggest that transcription and ARID1A both strongly promote the initial, transient recruitment of RAD52 to DNA damage, and thus likely act in a step upstream of RAD52. However, the longer-term, more stable RAD52 recruitment is only dependent on transcription in G1 cell cycle phase and strongly depends on ARID1A activity and DNA end-resection by EXO1 (Table 1).

**Table 1.** Characterization of biphasic RAD52 accumulation

	1st wave		2nd wave	
Cell cycle phase	G1	S/G2	G1	S/G2
Transcription dependent	yes	yes	yes	no
ARID1A dependent	yes	yes	yes	yes
EXO1 dependent	no	no	yes	yes

### ARID1A promotes RNaseH1 recruitment and R-loop resolution

**Figure 6.**
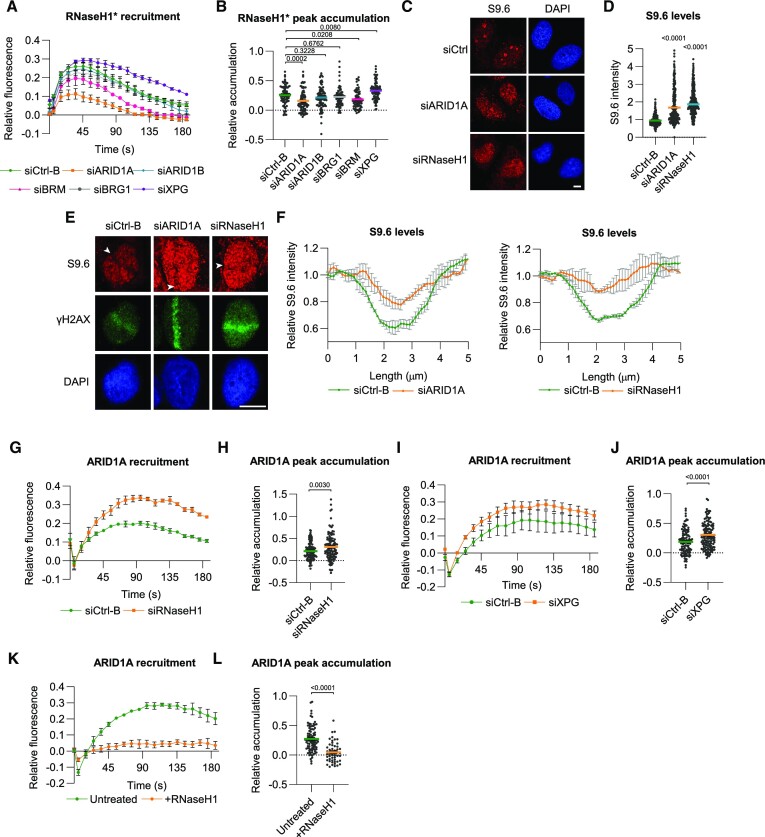
ARID1A promotes RNaseH1-mediated R-loop resolution near DNA breaks. (**A**) Quantification of real-time GFP-RNaseH1(D210N) (mutation indicated with *) recruitment to laser tracks in U2OS cells treated with control, ARID1A, ARID1B, BRG1, BRM and XPG siRNAs. Mean and SEM of three independent experiments. (**B**) Quantification of peak accumulation (35–45 s) in experiments shown in (A). Cells were pooled and for siCtrl-B *n* = 96, siARID1A *n* = 68, siARID1B *n* = 99, siBRG1 *n* = 82, BRM n = 72 and siXPG *n* = 86. (**C**) Representative immunofluorescence images showing S9.6 RNA-DNA hybrid staining in control, ARID1A and RNaseH1-depleted MRC-5 cells in unperturbed conditions. DNA is stained using DAPI. (**D**) Quantification of nuclear S9.6 signal intensity in immunofluorescence experiments in MRC-5 cells as depicted in (C). Pooled cells from three independent experiments where siCtrl-B *n* = 391, siARID1A *n* = 373 and siRNaseH1 *n* = 404. (**E**) Representative immunofluorescence images showing S9.6 RNA–DNA hybrid staining in laser-irradiated MRC-5 cells treated with control, ARID1A or RNaseH1 siRNA. Cells were fixed 1 min after laser irradiation. γH2AX staining is used as DNA damage marker and DNA is stained using DAPI. (**F**) Quantification of S9.6 signal intensity along a line perpendicular to the laser-induced DNA damage track marked by γH2AX in immunofluorescence experiments in MCR-5 cells as depicted in (E). The image shows the mean and SEM of three independent experiments. (**G**) Quantification of real-time ARID1A-mAID-mClover recruitment to laser-tracks in HCT116 treated with control or RNaseH1 siRNAs. Mean and SEM of three independent experiments. (**H**) Quantification of peak accumulation (85–105 s) in experiments shown in (G). Cells were pooled and for siCtrl *n* = 132 and siRNaseH1 *n* = 113. (**I**) Quantification of real-time ARID1A-mAID-mClover recruitment to laser-tracks in HCT116 cells treated with control or XPG siRNAs. Mean and SEM of three independent experiments. (**J**) Quantification of peak accumulation (85–105 s) in experiments shown in i. Cells were pooled and for siCtrl *n* = 169 and XPG *n* = 161. (**K**) Quantification of real-time ARID1A-mAID-mClover recruitment to laser-tracks in HCT116 cells untreated or overexpressing RNaseH1-mCherry. Mean and SEM of three independent experiments. (**L**) Quantification of peak accumulation (85–105 s) in experiments shown in (K). Cells were pooled and for untreated *n* = 92 and RNaseH1-mCherry overexpression n = 52. For quantification of the DNA damage recruitment, the relative fluorescence, corrected for background signal, was measured over time in the DNA damage tracks and normalized to the pre-damage fluorescence intensity. In each graph, numbers indicate *P* values obtained using an unpaired t-test (in H, J, L) or one-way ANOVA test (in B, D). Scale bar, 10 μm.

Together with XPG, transcription-dependent RAD52 is implicated in resolving R-loops that arise during DSB formation in transcribed genes (8,11,20,22,23,63). Because RAD52 recruitment is compromised in ARID1A-depleted cells, we wondered whether SWI/SNF complexes might regulate R-loop processing near DSBs. To study this, we first used U2OS cells expressing a doxycycline-inducible GFP-tagged inactive RNaseH1 mutant (D210N), which exhibits prolonged R-loop binding and can therefore be used as live cell marker to monitor R-loop formation (64). We observed that the GFP-RNaseH1 mutant was rapidly and transiently recruited to laser-induced DNA damage, which was more sustained after depletion of XPG (Figure 6A and B), confirming the formation of R-loops that are processed by XPG at DSBs (65). Interestingly, ARID1A depletion led to reduced RNaseH1 recruitment (Figure 6A and B). This was also observed after BRM depletion, albeit to a lesser extent, but not after depletion of ARID1B or BRG1. These results either suggest that ARID1A, possibly in complex with BRM, acts upstream of and promotes RNaseH1 recruitment to resolve R-loops or that ARID1A activity facilitates R-loop formation itself.

To distinguish between these two possibilities, we performed immunofluorescence in MRC-5 cells using the S9.6 antibody that specifically detects RNA–DNA hybrids (66). We used MRC-5 cells as our S9.6 R-loop staining protocol had been optimized in these fibroblasts. A previous study had already suggested that the absence of SWI/SNF subunits leads to increased R-loop formation in unperturbed cells (67). Indeed, we found that cells with ARID1A depletion had an overall higher S9.6 nuclear signal, similar as after RNaseH1 depletion (Figure 6C and D). Subsequently, we measured R-loop levels in multiphoton laser tracks, in cells fixed both 1 min and 1 h after laser irradiation. Strikingly, this showed that in cells fixed 1 min after DNA damage induction, R-loop levels drop substantially in the damaged area (Figure 6E, F), which is still visible 1 h after damage induction (Supplementary Figure S3K–M). This is in line with the rapid and transient recruitment of RNaseH1 and likely indicates that R-loops are swiftly removed at DSB sites to allow repair (11,20,22). However, cells depleted of ARID1A or RNaseH1 retained higher R-loop levels in the damaged area, clearly visible in cells fixed 1 min (Figure 6E and F), as well as 1 h after DNA damage induction (Supplementary Figure S3K–M). These results therefore indicate that ARID1A does not promote R-loop formation but their removal.

Subsequently, we studied whether the accumulation of ARID1A itself was R-loop dependent. We found that ARID1A recruitment to laser-induced DNA damage was increased in conditions of more R-loops, i.e. upon depletion of RNaseH1 (Figure 6G and H) or XPG (Figure 6I and J). In contrast, ARID1A recruitment was strongly inhibited in conditions with less R-loops, i.e. after transient overexpression of mCherry-tagged RNaseH1 (Figure 6K and L). These results indicate that R-loop formation is necessary for efficient ARID1A recruitment to DSBs in transcribing genes. We therefore conclude that there may be a feedback loop in which ARID1A-containing BAF complexes are recruited to DSB sites promoted by R-loop formation, after which ARID1A promotes the recruitment of RNaseH1, either directly or in an upstream step, to help process these R-loops to allow DNA repair.

### PBAF and ncBAF regulate transcription by promoting RNA polymerase II eviction which is maintained by BAF

**Figure 7.**
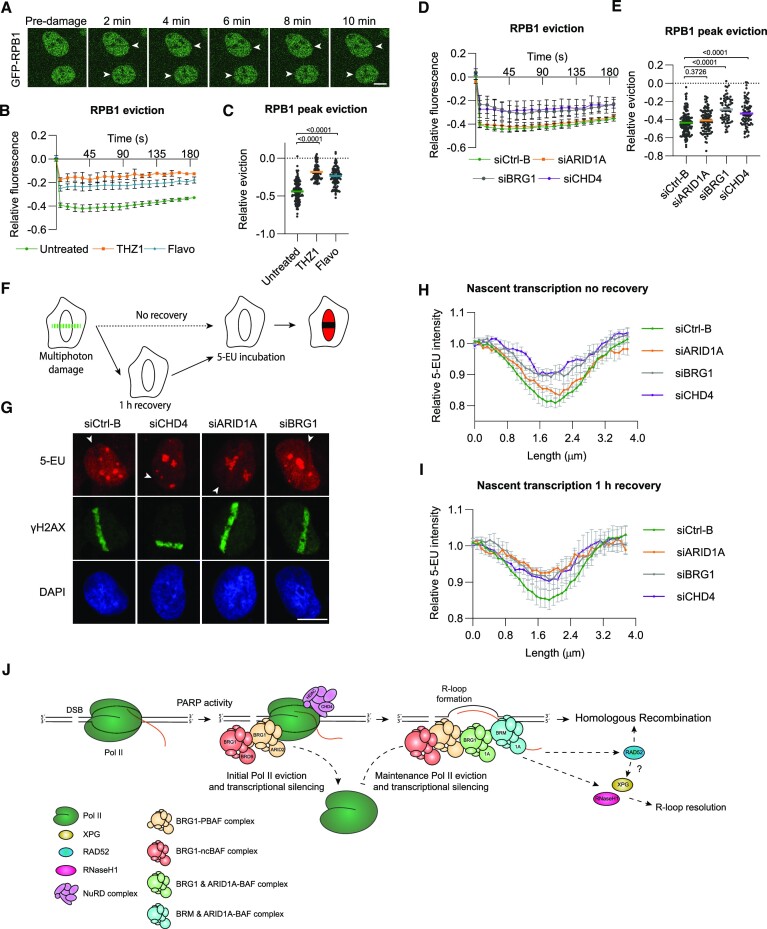
PBAF, ncBAF and BAF complexes initiate and maintain transcriptional silencing by promoting RNA polymerase II eviction. (**A**) Representative images of real-time GFP-RPB1 eviction in laser-generated DNA damage tracks in MRC-5 cells. Arrowheads indicate where the DNA damage was induced. (**B**) Quantification of real-time GFP-RPB1 eviction in laser-tracks in MRC-5 cells untreated or treated with 1 μM THZ1 or 1 μM flavopiridol (flavo). Mean and SEM of three (flavo) or two (THZ1) independent experiments. (**C**) Quantification of eviction (35–55 s) in experiments shown in (B). Cells were pooled and for untreated *n* = 164, THZ1 *n* = 85 and Flavopiridol *n* = 101. (**D**) Quantification of real-time GFP-RPB1 eviction in laser-tracks in MRC-5 cells treated with control, ARID1A, BRG1 or CHD4 siRNA. Mean and SEM of three independent experiments. (**E**) Quantification of eviction (35–55 s) in experiments shown in (D). Cells were pooled and for siCtrl-B *n* = 92, siARID1A *n* = 99, siBRG1 *n* = 64 and siCHD4 *n* = 77. (**F**) Scheme showing the assay used to monitor nascent transcription by visualizing 5-ethynyl uridine (5-EU) incorporation. Following multiphoton laser-irradiation cells were immediately incubated with 5-EU (no recovery) or after a 1 h recovery period. (**G**) Representative images of laser-irradiated U2OS cells transfected with the indicated siRNAs and incubated with 5-EU immediately after damage induction. Nascent transcription is visualized by labeling 5-EU with Atto 594 and DNA damage by staining for γH2AX. DNA is stained using DAPI. Arrowheads indicate where the DNA damage was induced. (**H**) Quantification of nascent transcription levels immediately after damage along a line perpendicular to the laser-induced DNA damage track marked by γH2AX as shown in (**G**). siCtrl-B, siARID1A, siBRG1 and siCHD4-treated U2OS cells were incubated with 5-EU immediately after damage induction. The image shows the mean and SEM of four (siCtrl-B, siARID1A and siBRG1) or three (siCHD4) independent experiments. **(I)** Quantification of nascent transcription levels 1 h after damage along a line perpendicular to the laser-induced DNA damage track marked by γH2AX as shown in Supplementary Figure S5G. siCtrl-B, siARID1A, siBRG1 and siCHD4-treated U2OS cells were incubated with 5-EU 1 h after damage induction. The image shows the mean and SEM of four (siCtrl, siBRG1) or three (siARID1A, siCHD4) independent experiments. (**J**) Model of the multiple functions of SWI/SNF during transcription-coupled homologous recombination. Upon DSB formation in active genes, PARP-dependent signaling recruits the NuRD and BRG1-containing PBAF and ncBAF complexes, which promote Pol II eviction and initial transcriptional silencing. Different BAF complexes are recruited in a PARP-, NuRD and R-loop-dependent manner to facilitate (i) maintenance of Pol II eviction and transcriptional silencing by a BRG1/ARID1A-BAF complex; (ii) RNaseH1 recruitment to resolve R-loops by a BRM/ARID1A-BAF complex and (iii) RAD52 accumulation to promote HR by an ARID1A-containing BAF complex. For quantification of the DNA damage eviction, the relative fluorescence, corrected for background signal, was measured over time in the DNA damage tracks and normalized to the pre-damage fluorescence intensity. In each graph, numbers indicate p values obtained using a one-way ANOVA test (in E, C). Scale bar, 10 μm.

Previously, both CHD4, as part of the NuRD complex, and BRG1, as part of the PBAF complex, have been implicated in mediating transcriptional silencing at DSB sites (14,15). To study whether ARID1A, ARID1B, BRM and BRG1, as part of different BAF, PBAF or ncBAF complexes, are involved in this process, we used previously generated MRC-5 cells expressing fluorescent Pol II due to knock-in of GFP at the endogenous locus of the main catalytic subunit RPB1 (68). Introducing DSBs with multiphoton laser led to an immediate and persistent eviction of GFP-RPB1 from chromatin, clearly visible and quantified by measuring the loss in fluorescence intensity of Pol II in the laser tracks (Figure 7A–C). Importantly, this eviction was dependent on transcription elongation, as determined using THZ1 and flavopiridol, indicating that it is actively transcribing Pol II that is evicted (Figure 7B and C). This eviction therefore conforms to loss of Ser2-phosphorylated Pol II and local repression of nascent transcription that has been observed in similar laser track experiments (15,16,55,69). Furthermore, Pol II eviction was independent of HDAC activity and slightly dependent on PARP activity, as determined using NaBu and PARPi (Supplementary Figure S4A and B). However, we did notice that PARP inhibition strongly reduced the width of the laser-track containing the evicted Pol II (Supplementary Figure S4C–E). A previous study concluded that PARP promotes chromatin expansion and spreading of chromatin remodeling and DDR factors in chromatin flanking DSB sites, based on a similar PARPi-induced reduction in the width of laser-tracks (47). Thus, it appears that Pol II eviction and local transcription repression itself happen largely independently from PARP, but that these phenomena spread throughout nearby chromatin in a PARP-dependent manner.

Subsequently, we studied which types of SWI/SNF complexes are involved in evicting transcribing Pol II from the chromatin after DSBs are induced. Depletion of CHD4 or BRG1 by siRNA reduced Pol II eviction in multiphoton laser tracks without having a major effect on chromatin spreading (Figure 7D and E and Supplementary Figure S4F). This is in line with their previously described role in silencing nascent transcription at DSB sites (14,15). However, we did not observe any difference in Pol II eviction when we depleted ARID1A, ARID1B or BRM (Figure 7D, E and Supplementary Figure S4G–J). We confirmed that BRM is expressed in MRC-5 cells (Supplementary Figure S4K), ruling out that the absence of an effect after siRNA is because BRM is not expressed in these cells. As BRG1 acts as catalytic subunit in BAF, PBAF and ncBAF complexes (26), we additionally depleted the PBAF-specific ARID2 and ncBAF-specific BRD9 subunits and, intriguingly, observed that depletion of both subunits reduced Pol II eviction (Supplementary Figure S4L and M). These results confirm that NuRD and BRG1-containing PBAF complexes facilitate transcriptional silencing at DSBs and indicate that they likely do this by promoting the eviction of Pol II from damaged chromatin. Moreover, these results implicate a BRG1-containing ncBAF complex in this process as well. The BAF complex, however, involving ARID1A, ARID1B and BRM, is not needed for this process.

To confirm that the eviction of Pol II results in local transcription repression, we pulse-labeled siRNA-treated MRC-5 cells with 5-ethynyl uridine (5-EU) to monitor nascent transcription after multiphoton microirradiation. First, we determined the immediate impact of DSBs on transcription by measuring EU-incorporation in laser tracks within minutes after inducing DNA damage (‘no recovery’ in Figure 7F). We observed that transcription was immediately repressed, in line with the rapid Pol II eviction. Depletion of CHD4 and BRG1, ARID2 and BRD9 reduced this repression, confirming their role in mediating this transcriptional silencing after DNA damage (Figure 7G and H; Supplementary Figure S5A and B). Again, we did not find that ARID1A, ARID1B or BRM affected this process, as their depletion had no effect on transcriptional silencing at DSBs (Figure 7G, H and Supplementary Figure S5C, D). Subsequently, we measured transcription 1 h after laser microirradiation (‘1 h recovery’ in Figure 7F) and found that it was still repressed and that this was again dependent on CHD4, BRG1, ARID2 and BRD9 (Figure 7I and Supplementary Figure S5E and F). However, surprisingly, we noticed that at this timepoint also ARID1A depletion reduced transcription repression, while depletion of ARID1B and BRM still had no effect (Figure 7I and Supplementary Figure S5G and H). These results confirm that the NuRD and BRG1-containing PBAF and ncBAF complexes mediate transcriptional silencing at DSB sites and furthermore suggest that ARID1A, probably in a BRG1-containing BAF complex, is involved in maintaining this transcriptional silencing after DSB.

To furthermore confirm this, we again measured GFP-RPB1 eviction at laser-induced DSBs, but imaged for longer time periods. Interestingly, we observed that RPB1 eviction from damaged chromatin persisted for at least 8 hours after DNA damage induction in cells treated with control siRNA (Supplementary Figure S6). However, in cells in which BRG1 or ARID1A was depleted, RPB1 eviction was reversed, within, respectively, ∼24 min and an hour. These results therefore suggest that ARID1A-containing BAF complexes maintain transcriptional silencing by promoting the sustained eviction of Pol II from damaged chromatin.

## DISCUSSION

Here, we show that multiple different SWI/SNF complexes, i.e. BAF complexes that either contain BRM or BRG1 and ARID1A or ARID1B and PBAF and ncBAF complexes containing BRG1, function to promote HR in a transcription-dependent manner (Figure 7J). In combination with other studies highlighting different aspects of the transcription-coupled HR mechanism, our results suggest that upon DSB formation in active genes, the NuRD and BRG1-containing PBAF and ncBAF complexes are rapidly recruited in a PARP-dependent manner to induce Pol II eviction and transcriptional silencing in the vicinity of the DSB (13–15,55). PARP, the NuRD complex and R-loop formation furthermore promote the recruitment of ARID1A-containing BAF complexes that facilitate RAD52 (and XPG) recruitment to DSBs (8,36). Also, ARID1B-containing BAF complexes are recruited depending on PARP and HDAC activity, but we have not studied their function in detail. Particularly, an ARID1A- and BRM-containing BAF complex facilitates R-loop processing, by promoting RNaseH1 recruitment. Furthermore, an ARID1A- and BRG1-containing BAF complex helps to maintain Pol II eviction and transcriptional silencing for longer periods of time (Figure 7J). It should be noted that the reduced RAD52 recruitment, R-loop processing, Pol II eviction and transcriptional silencing observed after depletion of SWI/SNF subunits was often only partial, suggesting that additional factors likely promote these processes as well.

Our results suggest that multiple events, i.e. transcriptional silencing, R-loop processing and DNA repair, take place at DSBs in transcriptionally active DNA, in which different types of SWI/SNF complexes are involved. Transcriptional silencing near DSBs is thought to be important to promote efficient DNA repair and prevent genomic instability (13,70). Previously, using an inducible reporter gene and nascent transcription measurements, the PBAF subunits BRG1, ARID2/BAF200 and PBRM1/BAF180 were found to mediate transcriptional silencing near DSBs (13,14). Here, we confirm these results and, in addition, show that elongating RPB1 is rapidly evicted from DSB sites in a PBAF- and NuRD-dependent manner, which could be a mechanism to establish and/or maintain transcriptional silencing (13–19). Our results also implicate a BRG1-and BRD9-containing ncBAF complex in this process. Furthermore, we identify an additional layer of control, showing that specifically ARID1A and BRG1, and thus a BAF complex, are needed to maintain Pol II eviction and transcriptional silencing at DSB sites for long periods of time (>1 h after DNA damage induction). Upon laser-induced DSB induction, Pol II is instantly, i.e. within seconds, evicted from the damaged chromatin. Intriguingly, we found that PARP activity was not so much involved in this initial Pol II eviction, but more in the local chromatin spreading of this eviction. However, PARP activity is needed for the DSB recruitment of BRG1 and other factors previously implicated in establishing transcriptional silencing, such as the NELF and NuRD complexes, the histone demethylase KDM5A and the chromodomain Y-like CDYL1 protein (15,19,71,72). This could therefore imply that initial Pol II eviction and transcription shutdown occur before or separate from the recruitment of these factors. Nevertheless, PARP was found to be required for eviction of elongating Pol II observed in cells fixed 20 min after DNA damage induction (55). Thus, possibly following the initial, rapid eviction of Pol II, PARP activity is needed to recruit SWI/SNF complexes and other silencing factors to spread and then further establish Pol II eviction and transcriptional silencing. When DSBs occur in genes, this process also involves DNA-PK-dependent Pol II eviction by degradation, triggered by WWP2-mediated ubiquitylation of RPB1 (16,17). More research is needed to investigate how all these different factors act together and in concert with different SWI/SNF factors and histone modifications to regulate transcriptional activity near DSBs.

R-loops are likely formed as a direct result of Pol II eviction and transcription shutdown at DSBs, but RNA-DNA hybrids have also been proposed to be formed by *de novo* RNA synthesis at DSBs (20). Although R-loops appear to play a role in recruitment of certain repair factors, including ARID1A as we show here, eventually they need to be resolved to allow proper HR via RAD51 loading (20,22,63,73,74). We observed rapid ARID1A- and BRM-dependent recruitment of mutant RNaseH1, which indicates that R-loops are immediately formed but also immediately processed, in line with previous findings (24). Indeed, S9.6 staining showed that R-loops formed at DNA breaks are resolved within minutes in an ARID1A- and RNaseH1-dependent manner. It was shown that in unperturbed cells lacking SWI/SNF factors such as BRG1 and ARID1A, R-loop levels are elevated, contributing to transcription-replication conflicts and causing increased genomic instability (67,75). We also noticed elevated R-loop levels in unperturbed ARID1A-depleted cells, but found that the processing of R-loops formed in reaction to DNA damage is also impaired in absence of ARID1A. Mechanistically, this may be because ARID1A, likely in a BAF complex together with BRM, directly or in an upstream step, promotes the recruitment of RNaseH1. It will be interesting to determine whether also in unperturbed conditions SWI/SNF activity similarly facilitates RNaseH1 recruitment to R-loops.

In line with previous findings for ARID1A, BRG1 and ARID1/BAF200 (35,36,40,76), we found that ARID1A, ARID1B, BRG1 and BRM all promote DNA end resection and RAD51 loading. Several studies have indicated that in active genes, RAD51 loading is transcription- and RAD52-dependent (7,8,10,21,77), pointing to a transcription-dependent HR pathway mediated by RAD52. We found that ARID1A, and thus a BAF complex, acts upstream of RAD52 and facilitates its transcription-dependent recruitment to DSBs, both in S/G2 as well as G1 phase cells. Interestingly, we noticed that RAD52 was recruited in a biphasic manner to laser-induced DSBs, showing a rapid resection-independent first wave and a slower, but more persistent, resection-dependent second wave. The first wave was dependent on transcription, as also noted before (8,10) and correlates to the rapid recruitment observed for SWI/SNF factors, RNaseH1 and other transcription-dependent repair factors such as the NuRD complex and WWP2 (15,16,24). As RAD52 was found to be recruited to DSBs in human cells by R-loops and to facilitate R-loop processing via XPG (8,62), it could be that ARID1A promotes R-loop processing via RAD52 and XPG as well (Figure 7J). It will be interesting to investigate this in more detail in future studies, and to determine how SWI/SNF chromatin remodeling and RAD52 cooperate to recruit factors such as RNaseH1 and XPG to deal with R-loops at laser-induced DSBs. The second RAD52 recruitment wave, which was never described before, was not affected by transcription inhibition in S/G2 phase cells but strongly dependent on EXO1. Extensive long-range resection by EXO1 is thought to especially promote the single-strand annealing (SSA) DSB repair pathway for which RAD52 is also essential (78,79). Thus, this second wave possibly reflects the activity of RAD52 in SSA. ARID1A strongly stimulated this second wave as well and also previously BRG1 was shown to stimulate long-term stable RAD52 recruitment to DNA damage (36). This, combined with the previous finding that depletion of ARID1A reduces SSA in a reporter assay (35), strongly suggest that a SWI/SNF BAF complex comprising BRG1 and ARID1A promotes SSA via RAD52.

Despite the large amount of evidence, including ours, showing that SWI/SNF complexes promote DSB repair, it is not yet entirely clear which precise chromatin remodeling activities are involved. Reduced MNase sensitivity and increased histone occupancy of damaged DNA after BRM or BRG1 depletion suggests that SWI/SNF promotes chromatin relaxation at DNA damage sites (39,80,81). Indeed, we also noticed that ARID1A and BRG1 slightly promoted the chromatin spreading of GFP-RPB1 eviction at lased-induced DNA damage tracks (Supplementary Figure S4F). Possibly, chromatin relaxation helps to promote the recruitment of repair factors. Moreover, SWI/SNF-induced Pol II eviction and transcription repression may furthermore promote repair by preventing Pol II and the transcription machinery to interfere with DNA-end resection and repair factors. However, it still needs to be addressed if the same type of chromatin remodeling activity that leads to chromatin relaxation is also responsible for inducing transcriptional silencing.

Understanding the roles and regulation of SWI/SNF complexes in transcription and DNA repair is important for understanding tumorigenesis and to be able to exploit SWI/SNF-associated vulnerabilities in cancer cells to improve current cancer therapy. Genes encoding subunits of the SWI/SNF chromatin-remodeling family, in particular ARID1A, are among the most frequently found mutated genes in many different types of human cancer (26,27,82). These mutations often affect SWI/SNF function and could therefore be utilized in a synthetic lethality therapeutic approach to specifically kill cancer cells. For instance, given the importance of SWI/SNF to HR, PARPi therapy is currently investigated in clinical trials of ARID1A mutated cancers (82). Our results suggest that a similar approach could work with other mutated SWI/SNF subunits as well. Furthermore, cancer cells that have lost the function of one specific SWI/SNF factor or complex can often compensate for this using other or aberrant SWI/SNF complexes or redundant mechanisms, as we have previously shown for BRM/BRG1-dependent transcriptional regulation of the DNA repair/transcription protein GTF2H1 (29,33). These backup mechanisms, on which cancer cells rely for viability, could also be considered suitable targets for cancer therapy. In this light, it would be interesting for future studies to focus on DNA repair mechanisms that act redundant to SWI/SNF complexes and that may become essential for cells to survive DNA damage, such as that inflicted by cancer chemotherapy, in the absence of specific SWI/SNF factors.

## Supplementary Material

gkad609_Supplemental_File

## Data Availability

The mass spectrometry proteomics data have been deposited to the ProteomeXchange Consortium via the PRIDE (83) partner repository with the dataset identifier PXD035140. Source data are provided with this paper. Any other data are available from the corresponding author upon reasonable request.
