# Supplementary material for: Different SWI/SNF complexes coordinately promote R-loop- and RAD52-dependent transcription-coupled homologous recombination
Source: Nucleic Acids Res. 2023 Jul 20;51(17):9055–74. doi: 10.1093/nar/gkad609 (PMC10516656; doi:10.1093/nar/gkad609)
Supplement: gkad609_Supplemental_File [file gkad609_supplemental_file.pdf]

Supplementary Materials for

Different SWI/SNF complexes coordinately promote R-loop- and RAD52-dependent transcription-coupled homologous recombination

Carlota Davó-Martínez, Angela Helfricht, Cristina Ribeiro-Silva, Anja Raams, Maria Tresini, Sidrit Uruci, Wiggert A. van Cappellen, Nitika Taneja, Jeroen A.A. Demmers, Alex Pines, Arjan F. Theil, Wim Vermeulen, Hannes Lans*

*Corresponding author. Email: w.lans@erasmusmc.nl

This PDF file includes:

Supplementary Text
Figs. S1 to S7
Tables S1 to S5

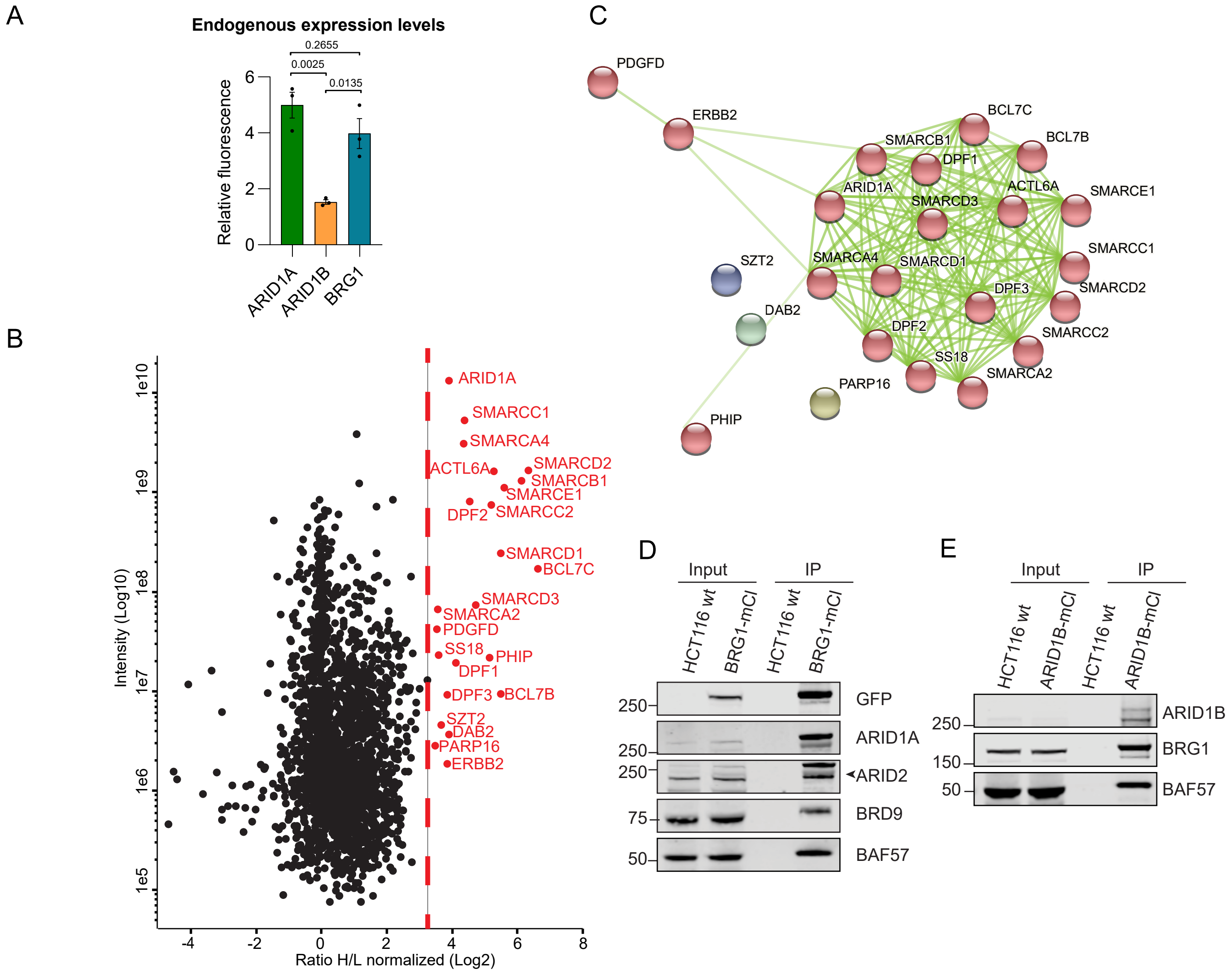

Supplementary Figure S1. Expression levels and interactions of endogenously tagged ARID1A, ARID1B and BRG1.

(**A**) Quantification of the relative fluorescence signal of mAID-mClover-tagged endogenous ARID1A, ARID1B and BRG1 proteins in HCT116 cells. Fluorescence signal was measured from live cell images from three independent experiments. (**B**) SILAC ratio plot (Log2) of ARID1A-mAID-mClover pull-down. Red dots indicate specific interactors of ARID1A determined by multidimensional significance (Benjamini-Hochberg FDR, Perseus software). The y axis represents total signal intensity of the ions associated with the identified amino acid sequence. (**C**) Protein interaction network based on the STRING database showing connections between ARID1A targets identified in SILAC shown in **b** with a medium confidence level of 0.4. (**D**) Immunoblot analysis of total cell lysate (Input) and mClover immunoprecipitation (IP) from parental HCT116 cells (HCT116 wt) and BRG1-mAID-mClover knock-in cells. Blot was stained with antibodies against GFP, ARID1A, ARID2, BRD9 and BAF57. (**E**) Immunoblot analysis of total cell lysate (Input) and mClover immunoprecipitation (IP) from parental HCT116 cells (HCT116 wt) and BRG1-mAID-mClover knock-in cells. Blot was stained with antibodies against ARID1B, BRG1 and BAF57.

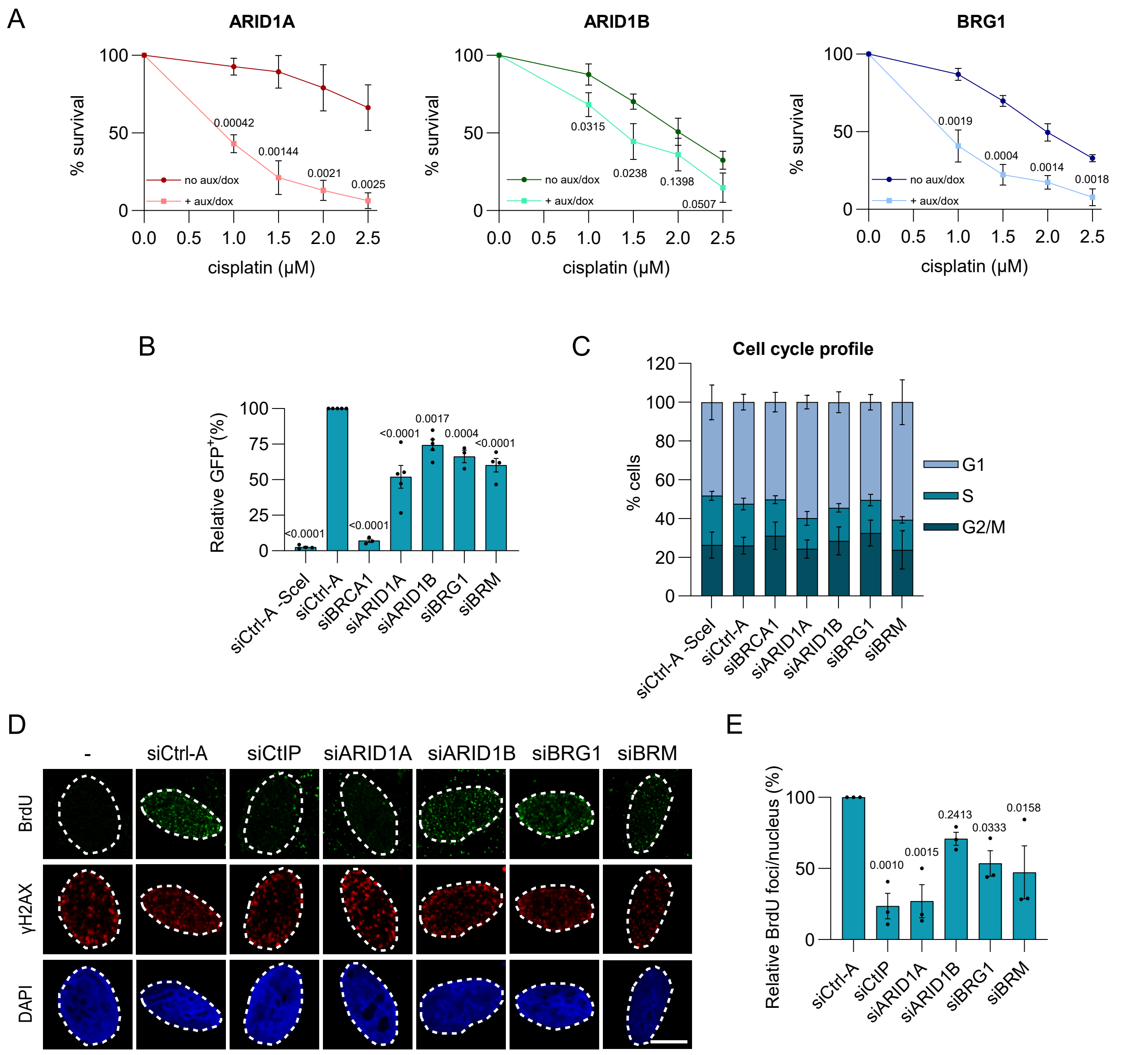

Supplementary Figure S2. SWI/SNF factors protect against cisplatin and promote DNA end resection.

(**A**) Cisplatin colony survival assay of ARID1A-, ARID1B- and BRG1-mAID-mClover knock-in HCT116 cells incubated with or without auxin and doxycycline (aux/dox). Mean and SEM of three independent experiments, each performed in triplicate. Numbers indicate p values obtained using an unpaired t-test. (**B**) Quantification of DR-GFP HR reporter assay showing the percentage of GFP positive cells upon double-strand break induction by I-SceI expression. Cells were transfected with the indicated siRNAs. Control siRNA treated cells without I-SceI expression (-SceI) were used as negative control. Mean and SEM of four (siCtrl-A, siARID1A/B, siBRM) or three (siBRG1, siBRCA1) independent experiments. Numbers indicate p values, which were obtained using an one-way ANOVA test. (**C**) Cell cycle distribution of U2OS cells (without (-SceI) or with I-SceI expression) treated with the indicated siRNAs, as determined by cell sorting of propidium iodide-stained cells. (**D**) Representative immunofluorescence images showing BrdU and γH2AX staining in U2OS cells labeled with BrdU and treated with the indicated siRNAs. Cells were treated for 3 h with neocarzinostatin prior to fixation. DNA is stained with DAPI. Scale bar, 10 μm. (**E**) Quantification of BrdU foci per nuclei in U2OS cells labeled with BrdU and treated with the indicated siRNAs and neocarzinostatin, as depicted in (**D**). Mean and SEM of three independent experiments. Numbers indicate p-values, as determined using a one-way ANOVA test.

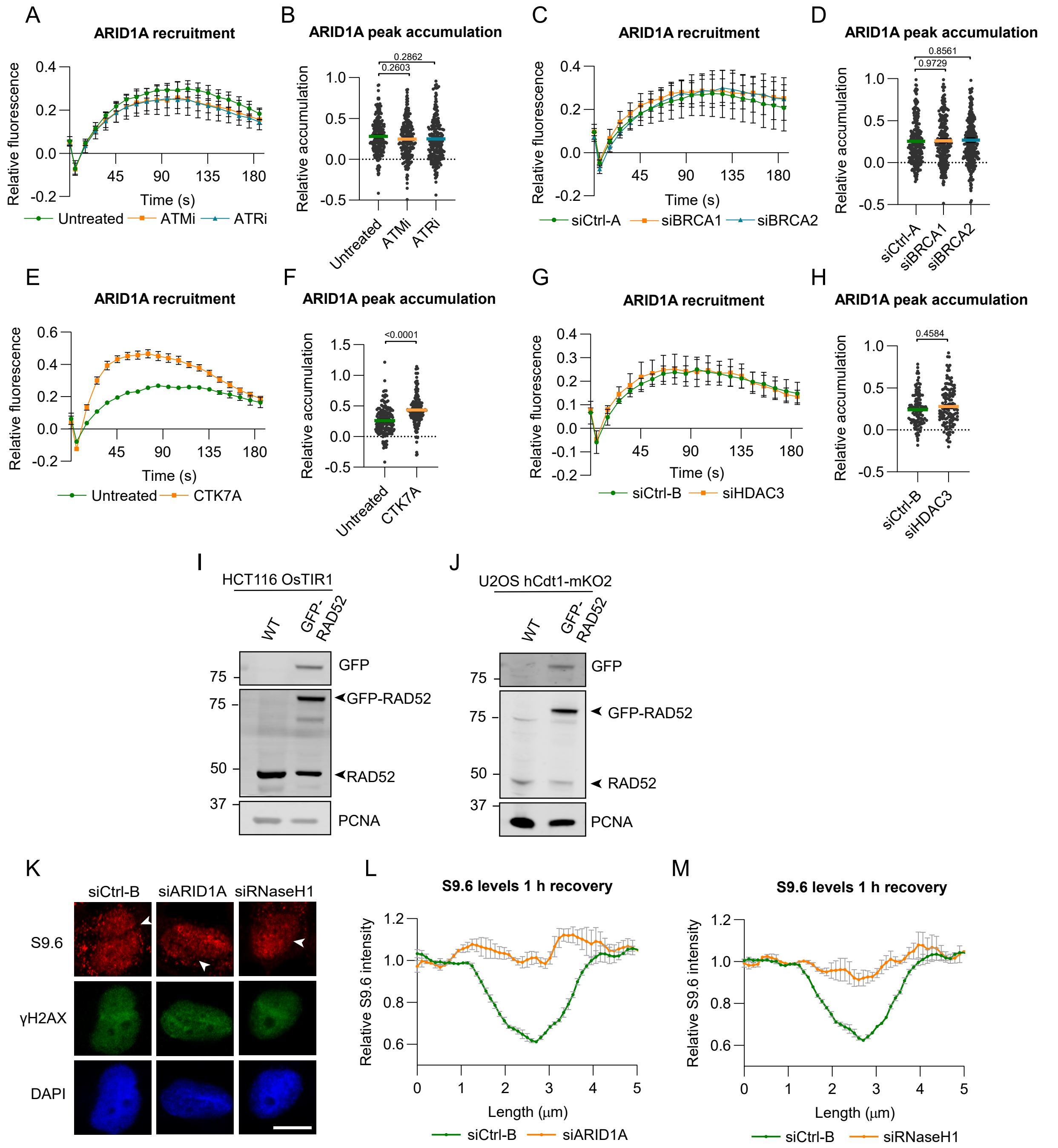

Supplementary Figure S3. ARID1A recruitment to double-strand breaks and R-loop resolution.
(**A**) Quantification of real-time ARID1A-mAID-mClover recruitment in HCT116 cells untreated or treated with 10 μM ATM (KU-55933) or ATR (Ceralasertib) inhibitor. Mean and SEM of three independent experiments. (**B**) Quantification of peak accumulation (85-105 s) in experiments shown in (**A**). Cells were pooled and for untreated n=240, ATMi n=236 and ATRi n=248. (**C**) Quantification of real-time ARID1A-mAID-mClover recruitment in HCT116 cells treated with control, BRCA1 and BRCA2 siRNAs. Mean and SEM of three independent experiments. (**D**) Quantification of peak accumulation (85-105 s) in experiments shown in **c.** Cells were pooled and for siCtrl n=308, siBRCA1 n=251 and siBRCA2 n=251. (**E**) Quantification of real-time ARID1A-mAID-mClover recruitment in HCT116 cells untreated or treated with 100 nM p300 inhibitor CTK7A. Mean and SEM of three independent experiments. (**F**) Quantification of peak accumulation (85-105 s) in experiments shown in **e**. Cells were pooled and for untreated n=166 and CTK7A n=189. (**G**) Quantification of real-time ARID1A-mAID-mClover recruitment in HCT116 cells treated with control and HDAC3 siRNA. Mean and SEM of three independent experiments. (**H**) Quantification of peak accumulation

(85-105 s) in experiments shown in (**G**). Cells were pooled and for siCtrl-B n=119 and siHDAC3 n=133. (**I**) **I**mmunoblot analysis of lysates of wild type and GFP-RAD52-expressing HCT116 cells. Blot was stained with antibodies against GFP, RAD52 and PCNA, as loading control. (**J**) **I**mmunoblot analysis of lysates of wild type and GFP-RAD52-expressing hCdt1-mKO2-transgenic U2OS cells. Blot was stained with antibodies against GFP, RAD52 and PCNA, as loading control. (**K**) Representative immunofluorescence images showing S9.6 RNA-DNA hybrid staining in laser-irradiated MRC-5 cells treated with control, ARID1A or RNaseH1 siRNA fixed 1 h after irradiation. γH2AX staining is used as DNA damage marker and DNA is stained using DAPI. (**L, M**) Quantification of S9.6 signal intensity along a line perpendicular to the laser-induced DNA damage track marked by γH2AX in immunofluorescence experiments in MCR-5 cells as depicted in (**K**). The image shows the mean and SEM three independent experiments. For quantification of the DNA damage recruitment, the relative fluorescence, corrected for background signal, was measured over time in the DNA damage tracks and normalized to the pre-damage fluorescence intensity. In each graph, numbers indicate p values obtained using a one-way ANOVA test (in **B**, **D**) or an unpaired t-test (in **F**, **H**).

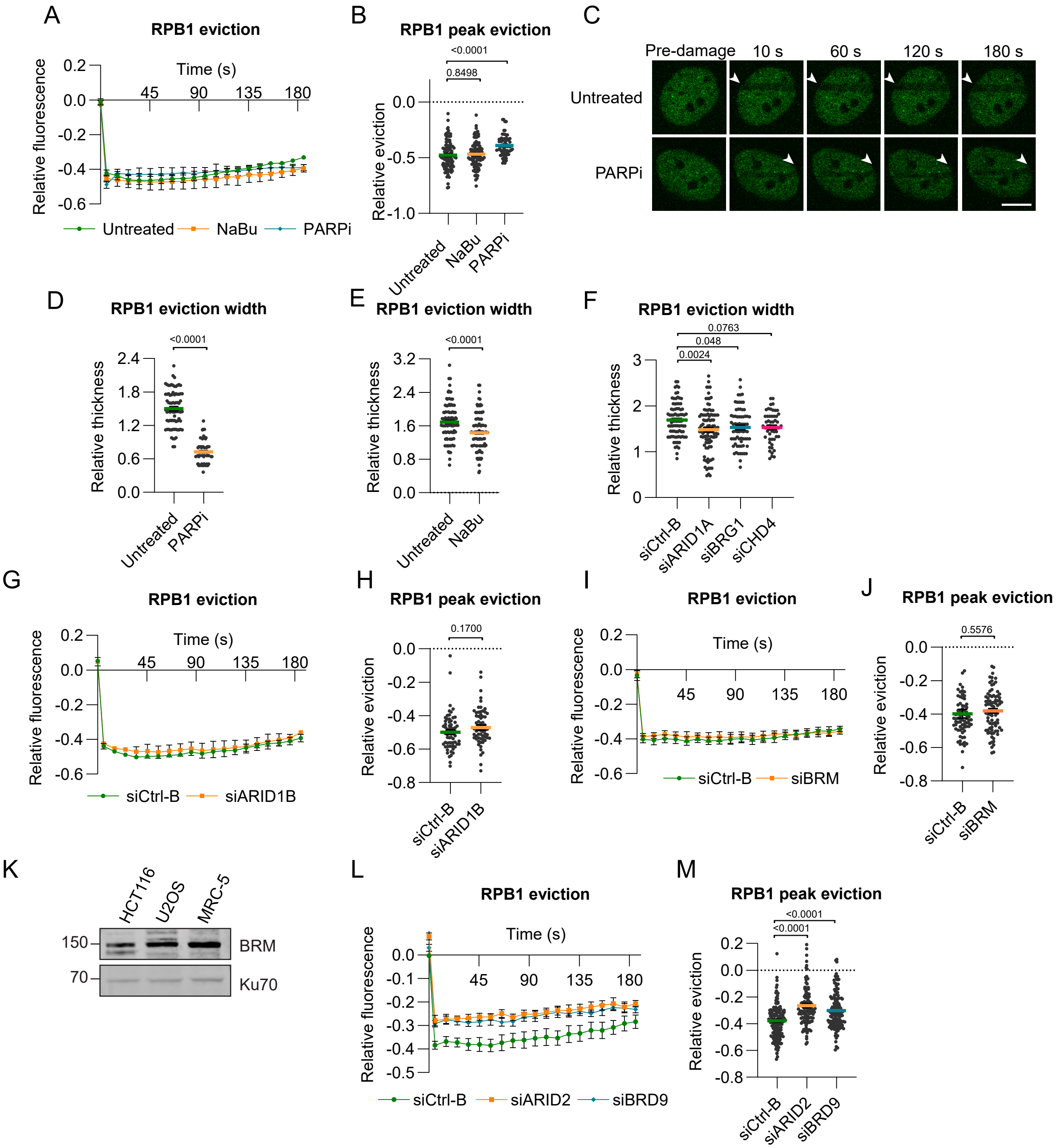

Supplementary Figure S4. RNA pol II eviction and transcriptional silencing in double-strand break laser tracks.

(**A**) Quantification of real-time GFP-RPB1 eviction in MRC-5 cells untreated or treated with 10 µM PARPi or 5 mM NaBu. Mean and SEM of three (untreated, NaBu) or two (PARPi) independent experiments. (**B**) Quantification of eviction (35-55 s) in experiments shown in **a.** Cells were pooled and for untreated n=164, PARPi n=82 and NaBu n=130. (**C**) Representative images showing real-time GFP-RPB1 eviction in laser-tracks in untreated and 10 µM PARPi-treated MRC-5 cells. Arrowheads indicate where the damage was induced. (**D, E, F**) Quantification of the width of the GFP-RPB1 eviction track in laser-irradiated MRC-5 cells untreated or treated with (**D**) 10 µM PARPi (untreated n=77 and PARPi n=106); (**E**) 5 mM NaBu (untreated n=134 and NaBu n=147); or (**F**) control, ARID1A, BRG1 and CHD4 siRNAs (siCtrl-B n=87, siARID1A n=97, siBRG1 n=69 and siCHD4 n=54). (**G**) Quantification of real-time GFP-RPB1 eviction in MRC-5 cells treated with control or ARID1B siRNA. Mean and SEM of two independent experiments. **(H)** Quantification of eviction (35-55 s) in experiments shown in (**G**). Cells were pooled and for siCtrl-B n=65 and siARID1B n=63.

(**I**) Quantification of real-time GFP-RPB1 eviction in MRC-5 cells treated with control or BRM siRNA. Mean and SEM of three independent experiments. (**J**) Quantification of eviction (35-55 s) in experiments shown in (**J**)**.** Cells were pooled and siCtrl-B n=74 and siBRM n=85. (**K**) Immunoblot analysis of HCT116, U2OS and MRC-5 cell lysate, staining with antibodies against BRM. Ku70 staining was used as loading control. (**L**) Quantification of real-time GFP-RPB1 eviction in MRC-5 cells treated with control, ARID2 or BRD9 siRNA. Mean and SEM of three independent experiments. (**M**) Quantification of eviction (35-55 s) in experiments shown in (**L**)**.** Cells were pooled and siCtrl-B n=175, siARID2=134, and BRD9 n=151. In each graph, numbers indicate p values obtained using one-way ANOVA test (in **B, F, M**) or an unpaired t-test (in **D, E, H, J**). Scale bar, 10 µm.

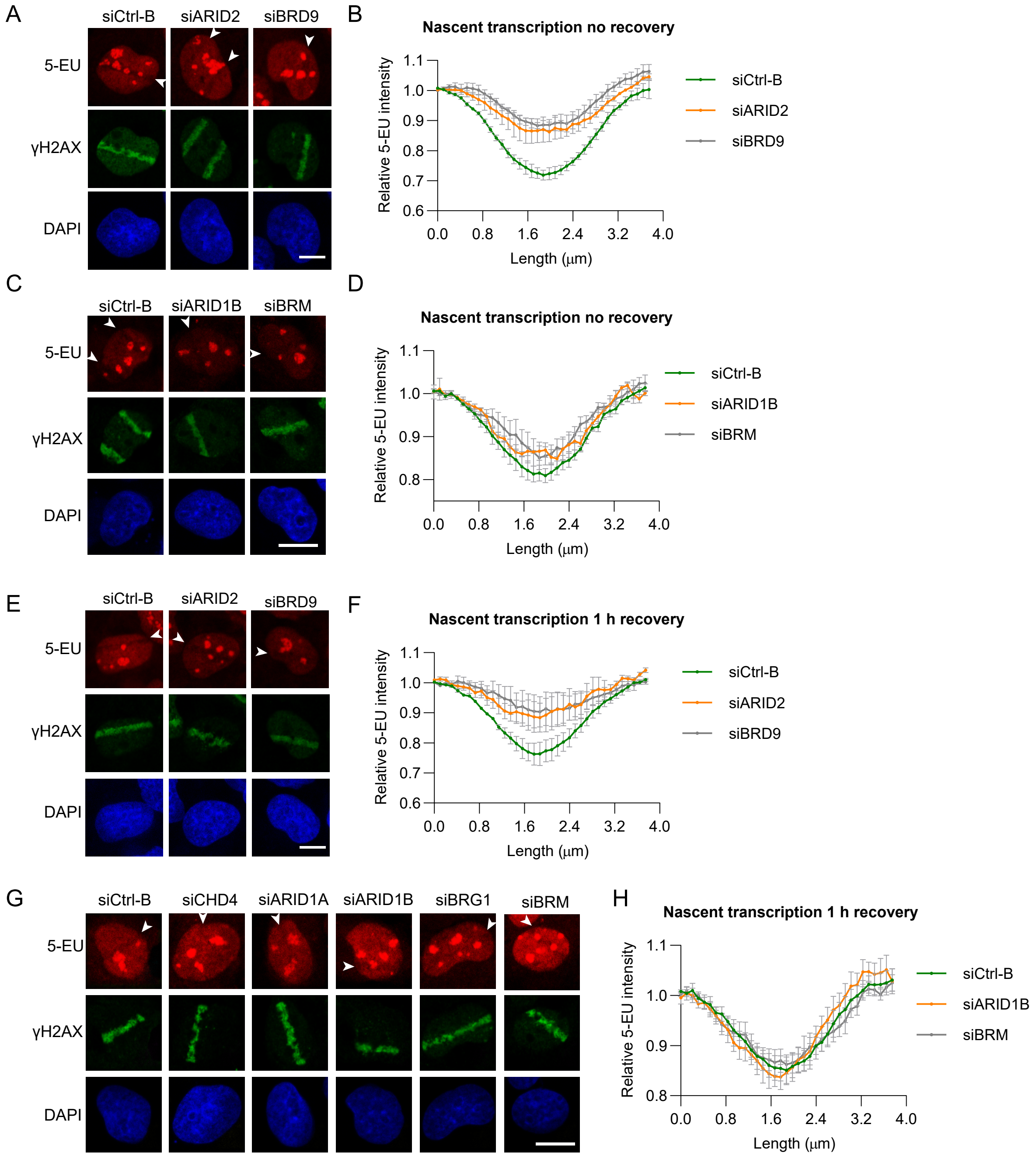

Supplementary Figure S5. PBAF and ncBAF complexes but not BAF complex are important for transcriptional silencing after DNA damage.

(**A**) Representative images of laser-irradiated U2OS cells transfected with the indicated siRNAs and incubated with 5-EU immediately after damage induction. Nascent transcription is visualized by labeling 5-EU with Atto 594 and DNA damage by staining for γH2AX. DNA is stained using DAPI. Arrowheads indicate where the DNA damage was induced. (**B**) Quantification of nascent transcription levels along a line perpendicular to the laser-induced DNA damage track marked by γH2AX as shown in (**A**). siCtrl-B, siARID2 and siBRD9-treated U2OS cells were incubated with 5-EU immediately after damage induction. The image shows the mean and SEM of three independent experiments. (**C**) Representative images of laser-irradiated U2OS cells transfected with the indicated siRNAs and incubated with 5-EU immediately after damage induction. Nascent transcription is visualized by labeling 5-EU with Atto 594 and DNA damage by staining for γH2AX. DNA is stained using DAPI. Arrowheads indicate where the DNA damage was induced. (**D**) Quantification of nascent transcription levels along a line perpendicular to the laser-induced DNA damage track marked by γH2AX as shown in (**C**). siCtrl-B, siARID1B and siBRM-treated U2OS cells were incubated

with 5-EU immediately after damage induction. The image shows the mean and SEM of four (siCtrl-B, siBRM) or three (siARID1B) independent experiments. (**E**) Representative images of laser-irradiated U2OS cells transfected with the indicated siRNAs and incubated with 5-EU 1 h after damage induction. (**F**) Quantification of nascent transcription levels along a line perpendicular to the laser-induced DNA damage track marked by γH2AX as shown in (**E**). siCtrl-B, ARID2 and siBRD9-treated U2OS cells were incubated with 5-EU 1 h after damage induction. The image shows the mean and SEM of three independent experiments (**G**) Representative images of laser-irradiated U2OS cells transfected with the indicated siRNAs and incubated with 5-EU 1 h after damage induction. (**H**) Quantification of nascent transcription levels along a line perpendicular to the laser-induced DNA damage track marked by γH2AX as shown in (**G**). siCtrl-B, siARID1B and siBRM-treated U2OS cells were incubated with 5-EU 1 h after damage induction. The image shows the mean and SEM of four independent experiments

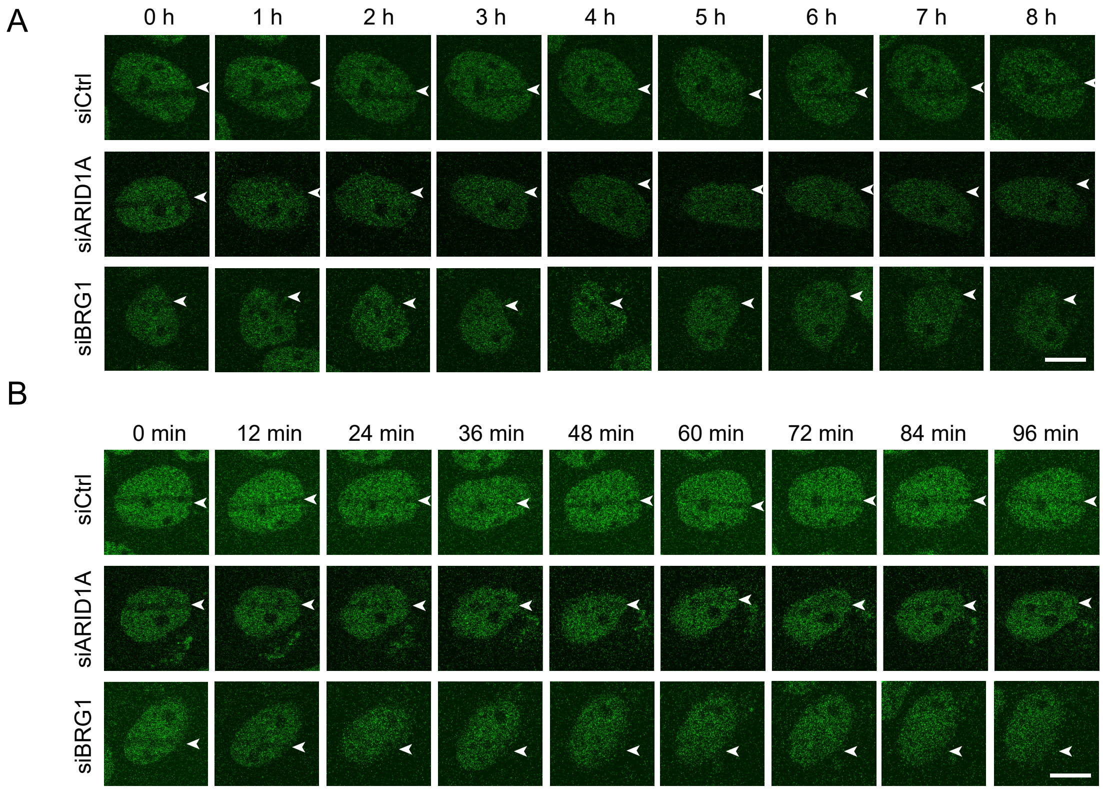

Supplementary Figure S6. ARID1A helps to maintain GFP-RPB1 eviction from DNA damage sites. (**A**) Representative images showing real-time GFP-RPB1 eviction from laser-induced DNA damage tracks in MRC-5 cells treated with control, ARID1A or BRG1 siRNA for eight hours. Scale bar, 10 μm. (**B**) Representative images showing real-time GFP-RPB1 eviction from laser-induced DNA damage tracks in MRC-5 cells treated with control, ARID1A or BRG1 siRNA for 96 minutes. Scale bar, 10 μm.

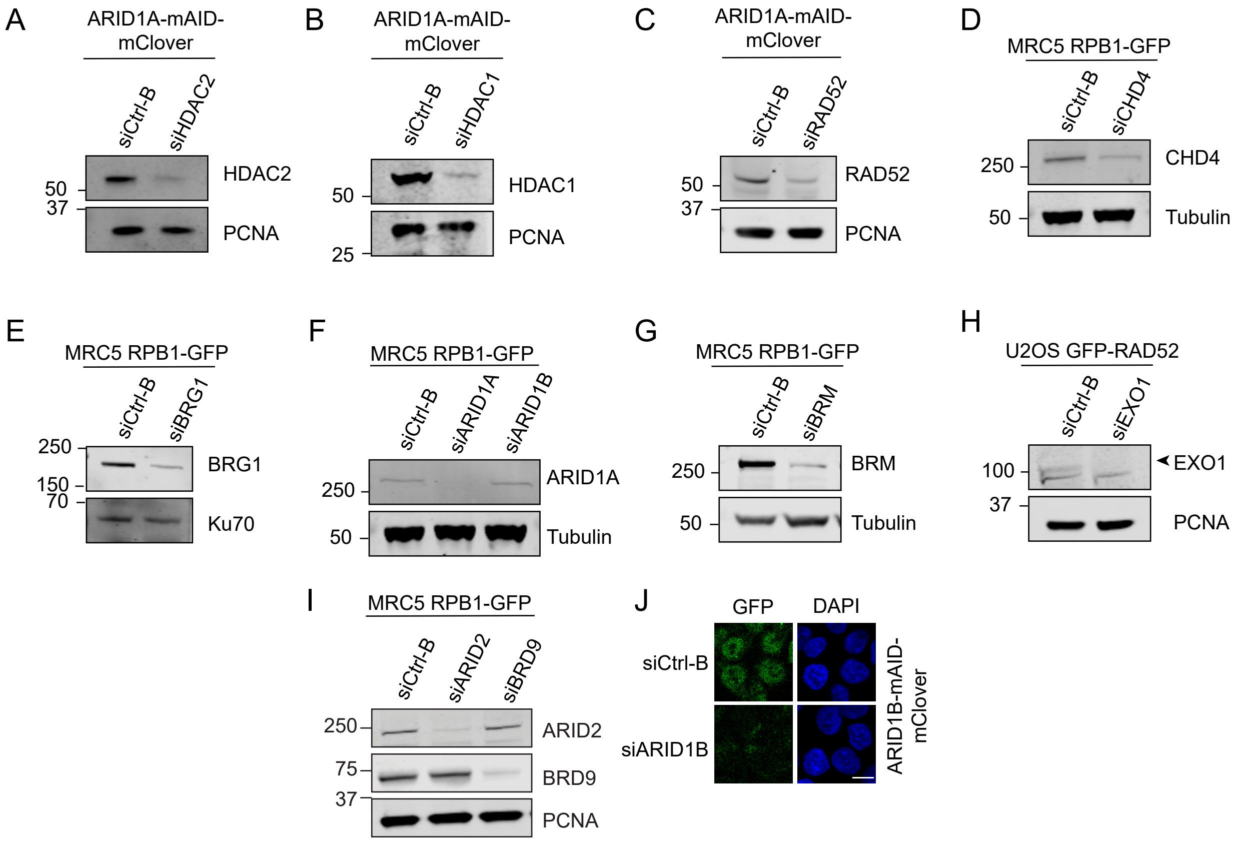

Supplementary Figure S7. siRNA-mediated protein depletion

(**A-I**) Immunoblot analyses of total cell lysates demonstrating the efficiency of siRNA-mediated protein depletion with siRNAs against HDAC1, HDAC2, RAD52, CHD4, BRG1, ARID1A, BRM, EXO1, ARID2 and BRD9 in the indicated cell lines and probed with antibodies against the respective proteins. Tubulin, Ku70 or PCNA were used as loading control. (**J**). Representative immunofluorescence image of protein depletion with siRNA against ARID1B in HCT116 ARID1B-mAID-mClover knock-in cells. Scale bar, 10 μm.

Supplementary Table S1.

Cell line	Source
HCT116 OsTIR1	Ref. 40
HCT116 OsTIR1 ARID1A-mAID-mClover	This paper
HCT116 OsTIR1 ARID1B-mAID-mClover	This paper
HCT116 OsTIR1 BRG1-mAID-mClover	This paper
HCT116 OsTIR1 GFP-RAD52	This paper
U2OS	ATCC
U2OS T-REx GFP-RNaseH1 D210N	Ref. 59
U2OS GFP-RAD52(AAVS1) + MKO2-Cdt1	This paper
U2OS DR-GFP	Ref. 80
MRC-5	ATCC
MRC-5 GFP-RPB1	Ref. 63
S9.6 HB-8730	ATCC

Cell lines used in this study.

Supplementary Table S2.

siRNA	Sequence
siARID1A	5'-GCAGGCACCACUAACUUAU-3'
siARID1B	5'-AACCGCUCGGAAUUCCUUU-3'
siARID2	5'-CAGUUUCACAGGGUCAACA-3'
siBRCA1	5'-GGAACCUGUCUCCACAAAG -3'
siBRCA2	5'-GAAGAAUGCAGGUUUAAUA-3'
siBRD9	5'- GCUUUAAGAUGAUGAGCAA -3'
siBRG1	5'-CCAAGCCGGUCGUGAGUGA-3'
siBRM	5'-GAAAGGAGGUGCUAAGACA-3'
siCHD4	5'-GAGCGGCAGUUCUUUGUGA-3'
siCtIP	5'-GCUAAAACAGGAACGAAUC-3'
siCtrl-A	5'-UGGUUUACAUGUCGACUAA-3'
siCtrl-B	5'-UGGUUUACAUGUUGUGUGA-3'
siEXO1	5'-GCACGUAAUUCAAGUGAUG-3'
siHDAC1	5'-CUAAUGAGCUUCCAUACAA-3'
siHDAC2	5'-GCGGAUAGCUUGUGAUGAA-3'
siHDAC3	5'-GCAUUGAUGACCAGAGUUA-3'
siRAD52	5'-GGAAAUAUGAUCCAUCUUA-3'
siRNaseH1	5'-GACAGUAUGUUUACGAUAA-3'
siXPG	5'-CAUGAAAUCUUGACUGAUA-3'

siRNAs used in this study.

Supplementary Table S3.

Inhibitor	Source, Reference	Concentration
5-bromo-2′-deoxyuridine (BrdU)	Sigma, B5002	30 µM
5-ethynyl-uridine (5-EU)	Axxora, JBS-CLK-N002-10	0.4 mM
ATMi	Selleckchem, S1092	10 µM
ATRi	Selleckchem, S7693	10 µM
ATTO 594 AZIDE	Atto Tec, AD 594-105	60 µM
Auxin	Sigma, I3750	100 nM
CPTH2	Sigma, c9873	50 nM
CTK7A	Calbiochem, 382115	100 nM
Doxycycline	Sigma, D9891	40 ng/ml
Flavopiridol	Sigma F3055	1 µM
GSK126	Selleckchem, S7061	1 µM
NaBu	Sigma, B_5887	5 mM
Neocarzinostatin	Merck, N9162	200 ng/ml
PARPi	Axon Medchem, KU0058948	10 µM
THZ1	Xcessbio, M60214-2s	1 µM
TSA	Sigma, T8552	1 µM

Chemicals used in this study .

Supplementary Table S4.

Source, Reference	Antibody	Dilutions	
		Immunoblotting	Immunofluorescence
Abcam, ab11174	μH2AX	N.A.	1/1000
Santacruz, sc-32761	ARID1A	1/1000	N.A.
Abcam, ab57461	ARID1B	1/1000	N.A.
Santacruz, sc-166117	ARID2	1/1000	N.A.
Homemade, GR280	BAF57	1/1000	N.A.
Abcam, ab259839	BRD9	1/1000	N.A.
BD Biosciences, 555627	BrdU	N.A.	1/100
Abcam, ab110641	BRG1	1/10000	N.A.
Abcam, ab15597	BRM	1/800	N.A.
Abcam, ab72418	CHD4	1/5000	N.A.
Abcam, ab70163	CtIP	N.A.	1/1000
Abcam, ab155553	EXO1	1/1000	N.A.
Roche, 11814460001	GFP	1/1000	1/1000
Abcam, ab7029	HDAC2	1/5000	N.A.
Santacruz, sc-17789	Ku70	1/1000	N.A.
Abcam, ab50003	MDC1	N.A.	1/1000
Abcam, ab29 (PC10)	PCNA	1/1000	N.A.
Homemade #2307 (van Gent)	RAD51	N.A.	1/10000
Santacruz, sc-365341	RAD52	1/500	N.A.
Proteintech, 15606-1-AP	RNaseH1	1/2000	N.A.
Calbiochem, RPA34 ab-2	RPA34	N.A.	1/1000
Purified from S9.6 HB-8730	S9.6	N.A.	1/500
Sigma Aldrich, B512	Tubulin	1/5000	N.A.

Primary antibodies used in this study.

Supplementary Table S5.

Source, Reference	Antibody	Dilutions	
		Immunoblotting	Immunofluorescence
Sigma sab4600215	Anti-rabbit, CF IRDye 770	1/10000	N.A.
Sigma sab4600200	Anti-rabbit, CF IRDye 680	1/10000	N.A.
Sigma sab4600214	Anti-mouse, CF IRDye 770	1/10000	N.A.
Sigma sab4600199	Anti-mouse, CF IRDye 680	1/10000	N.A.
Invitrogen, A11034	Anti-rabbit, Alexa Fluor 488	N.A.	1/1000
Invitrogen, A11032	Anti-mouse, Alexa Fluor 594	N.A.	1/1000

Secondary antibodies used in this study.
